# The methodology of quantitative risk assessment studies

**DOI:** 10.1186/s12940-023-01039-x

**Published:** 2024-01-27

**Authors:** Maxime Rigaud, Jurgen Buekers, Jos Bessems, Xavier Basagaña, Sandrine Mathy, Mark Nieuwenhuijsen, Rémy Slama

**Affiliations:** 1 Inserm, University of Grenoble Alpes, CNRS, IAB, Team of Environmental Epidemiology Applied to Reproduction and Respiratory Health, Grenoble, France; 2 VITO, Flemish Institute for Technological Research, Unit Health, Mol, Belgium; 3 ISGlobal, Barcelona, 08003 Spain; 4 Universitat Pompeu Fabra (UPF), Barcelona, 08003 Spain; 5 CIBER Epidemiología y Salud Pública (CIBERESP), Madrid, 28029 Spain; 6 CNRS, University Grenoble Alpes, INRAe, Grenoble INP, GAEL, Grenoble, France

**Keywords:** Dose–response, Environment, Hazard, Health impact, Policy, Risk

## Abstract

Once an external factor has been deemed likely to influence human health and a dose response function is available, an assessment of its health impact or that of policies aimed at influencing this and possibly other factors in a specific population can be obtained through a quantitative risk assessment, or health impact assessment (HIA) study. The health impact is usually expressed as a number of disease cases or disability-adjusted life-years (DALYs) attributable to or expected from the exposure or policy. We review the methodology of quantitative risk assessment studies based on human data. The main steps of such studies include definition of counterfactual scenarios related to the exposure or policy, exposure(s) assessment, quantification of risks (usually relying on literature-based dose response functions), possibly economic assessment, followed by uncertainty analyses. We discuss issues and make recommendations relative to the accuracy and geographic scale at which factors are assessed, which can strongly influence the study results. If several factors are considered simultaneously, then correlation, mutual influences and possibly synergy between them should be taken into account. Gaps or issues in the methodology of quantitative risk assessment studies include 1) proposing a formal approach to the quantitative handling of the level of evidence regarding each exposure-health pair (essential to consider emerging factors); 2) contrasting risk assessment based on human dose–response functions with that relying on toxicological data; 3) clarification of terminology of health impact assessment and human-based risk assessment studies, which are actually very similar, and 4) other technical issues related to the simultaneous consideration of several factors, in particular when they are causally linked.

**Supplementary Information:**

The online version contains supplementary material available at 10.1186/s12940-023-01039-x.

## Introduction

The main aims of environmental health research are i) to identify positive or negative determinants of health-related states (environmental factors in a broad sense encompassing physical, chemical, social, behavioral, systemic factors); ii) to understand the mechanisms underlying the effects of these factors; iii) to quantify the corresponding population impact, which can either be a burden or benefit. This quantification can be done e.g., in terms of number of deaths or disease cases or of healthy years of life lost attributable to the factor or set of factors; and iv) to identify interventions (which can be of all natures and act e.g., on the body, behaviors, knowledge, representations, values, the social, physical and chemical environments, the economy) allowing to limit this impact and preserve or improve the health of populations and limit health inequalities.

In the classical view of the 1983 Redbook of the US National Research Council on risk assessment [1, 2], aim i) corresponds to hazard identification and aim iii) to risk assessment. Aim iv) is usually seen as being tackled by health impact assessment (HIA) studies, or analytical HIA studies [3] but as we will discuss (see "Issues related to terminology" below, last section), from a methodological point of view, the approaches used to tackle aims iii) and iv) are essentially similar. We will therefore use *(quantitative) risk assessment* to point to studies filling specifically the aim of steps iii) (quantification of population impact of existing factors) and iv) (which corresponds to the quantification of the expected impact of hypothetical policies or interventions). The overall aim of quantitative risk assessment as broadly defined can be described as the quantification of the population impact of any type of factor, exposure, policy or program, hypothesized or already present.

Risk assessment studies typically allow to answer questions such as “How many cases of these diseases are attributable (yesterday/today/tomorrow) to this (exposure) factor or policy?”, or “How many disease cases would be avoided today/in the future if this (exposure) factor was/had been brought to a certain level, or if this policy was/had been implemented, all other things not influenced by this factor or policy being kept identical?”. These questions relate to the consequences of interventions (more precisely about the comparison of counterfactual situations), and not about associations or effects (i.e., hazards, for example: can exposure to this factor cause liver cancer?), as is typical for epidemiological study designs such as cohorts and case–control studies. Such measures of associations, or dose–response functions, are essential but not sufficient to assess the risk. Indeed, dose–response functions alone generally do not allow providing a relevant hierarchy of the disease burden, or impact, associated with each factor: the impact can be higher for an exposure with a mild dose–response function curve than for an exposure with a steep dose–response function, if the first exposure is more frequent than the latter exposure.

For many, if not all risk factors that influence the occurrence of a health-related event, it is not possible to identify if the occurrence of this event in a given subject has been caused by the risk factor; indeed, disease causes generally do not leave a specific (unambiguous) signature in the body, even for strong associations such as the induction of lung cancer by active tobacco smoking. Therefore, one cannot add up cases identified from death certificates or other routine medical data to estimate the disease or mortality burden attributable to an exposure or policy. Similarly, *before-after* observational studies can be used to document quantitatively health changes in a given population, following e.g., a heat wave, a major air pollution episode [4], a decrease in air pollution following the temporary closure of an industrial site, an abrupt change in regulation [5] or an event such as the Olympic Games. However, they are no general solution here; indeed, they may allow to identify a hazard but they are limited to documenting observed (factual) changes and not to studying other (counterfactual) scenarios.

Consequently, one has to rely on more indirect – modelling – approaches. This can be done combining knowledge about the overall frequency of the health parameter considered in the specific population under study, about the distribution of exposure and about dose–response function(s) associated with the factor(s), typically stemming from long-term studies such as cohorts (or animal studies in the case of animal-based risk assessment approaches). Risk assessment studies are related to three research and activity streams: that of the epidemiological concept of population attributable fraction, or etiologic fraction, dating back from the mid-twentieth century [6–8], that of chemical risk assessment derived from toxicological studies [9], and the practice of environmental impact assessment in relation to a planned policy or project, in which the consideration of the health impacts possibly induced by the planned policy or project has become more frequent, in addition to the consideration of its environmental impacts [3, 10].

**Fig. 1:**
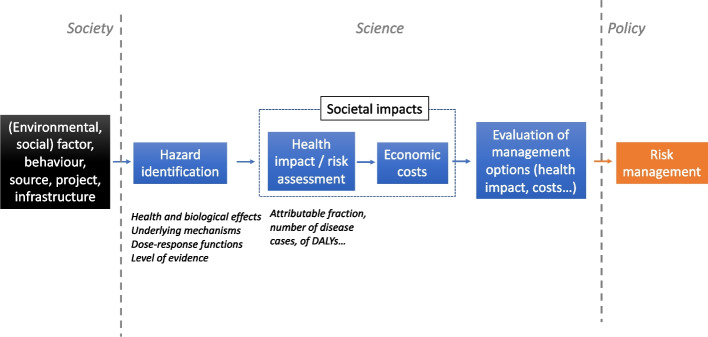
Position of quantitative risk assessment in the process of risk characterization and management. Risk assessment can be used to assess the impacts of the “factors” considered (leftmost box) and of policies aiming at managing and limiting their impacts. Adapted from [11]

These quantitative risk assessment studies contribute to integrating and translating knowledge generated from environmental health research in a form more relevant for policy making. They can be used to define or compare risk management strategies or projects, policies, infrastructures of various kinds with possible health consequences (Fig. 1). The risk assessment step can be followed by (or include) an economic assessment step, in which the estimated health impact is translated into an economic cost, providing an economic assessment of the impact of the factor or policy considered.

Many risk assessment studies have been conducted in relation to atmospheric pollutants, urban policies, metals, tobacco smoke, alcohol consumption, dietary factors, access to clean water. Although many chemicals are likely to influence health, the consideration of chemical exposures in (human-based) risk assessment studies appears relatively limited [12, 13]. The most recent worldwide environmental burden of disease assessment coordinated by the IHME (Institute for Health Metrics and Evaluation, Seattle, USA) considered 87 risk factors and combinations of risk factors, including air pollutants, non-optimal temperatures, lead, unsafe water, sanitation and handwashing, but no other chemical factors except those considered in an occupational setting [14]. However, the production volume of synthetic chemicals is still increasing and is expected to triple in 2050 compared to 2010 [15], and widespread human exposure is documented by biomonitoring studies [16, 17].

Several reviews about risk assessment and HIA studies have been published [10, 18–23]. For example, Harris-Roxas et al. provided a narrative of the historical origins of health impact assessment, strengths and opportunities [20], Nieuwenhuijsen [21] reviewed issues related to the participatory nature of HIA studies, while Briggs [19] provided a conceptual framework for integrated environmental health impact assessment. With the exception of issues related to the challenging concepts of etiologic fraction/excess cases [7, 8], very few reviews focused on methodological issues related to the technicalities of the execution of the assessment itself, while, with the development of more refined approaches to assess exposures, the identification of a growing number of hazards through toxicological and biomarker-based epidemiological studies or epidemiological studies based on fine-scale (e.g., air pollution) modeling, there is a need to review options and strategies related to input data and handling of uncertainties at each step of risk assessment studies.

We therefore aimed to perform a literature review (see [24]) of the methodology of quantitative risk assessment studies, discussing sequentially each step posterior to issue framing [19]. Qualitative health impact assessment approaches and those based on animal (toxicological) dose–response functions were not considered, with the exception of a few points illustrating in which respect the latter diverge from assessments based on human dose–response functions. We conclude by summarizing the identified methodological gaps, in particular related to the handling of emerging factors with partial data, and issues related to terminology.

## Key issues and options at each step

### Overall methodology of quantitative risk assessment studies

The main technical steps of quantitative risk assessment, include:

1. Definition/identification of the factor(s) (environmental factors/infrastructure/plan/policy) considered;
2. Definition of the study area and study population;
3. Description of the counterfactual situations compared and of the study period (may be merged with step 1);
4. Assessment/description of “exposures” in the study population under each counterfactual scenario;
5. Identification of the hazards (health outcomes) induced by the factors considered and of the corresponding dose–response functions and level of evidence;
6. Assessment of “baseline” (usually, current) disease frequency or of the DALYs attributable to the health outcomes considered, if needed;
7. Quantification of health risk or impact (e.g., in number of disease cases or DALYs);
8. Quantification of the social and economic impacts;
9. Uncertainty analysis;
10. Reporting/communication.

Note that some reviews [19, 25] include preparatory or organizational steps, which are not detailed here. Public involvement (not discussed here) can be present at virtually each of these steps. The steps of identification of the exposures, outcomes, area and population considered (numbered 1–3 above) and protocol definition are sometimes referred to as “scoping” step. Note also that the order of some steps is somewhat arbitrary. For example step 2 may come first.

Not all quantitative risk assessment studies follow all of these steps in practice, depending on their scope and the chosen approach. In practice, some studies may stop before completing the risk assessment step – for example many “risk assessment” exercises conducted by national agencies actually only correspond to the identification of hazards associated with the exposure or policy considered without quantification of the corresponding risk, e.g., because robust dose–response functions are lacking (see Sect. " Issues related to terminology" for further discussion). In case a policy is indeed implemented, a “policy implementation phase”, monitoring the implementation of the policy and possibly its actual impact, is sometimes added.

We will review steps 2 to 9, corresponding to the “appraisal steps” in the WHO terminology [25].

**Fig. 2:**
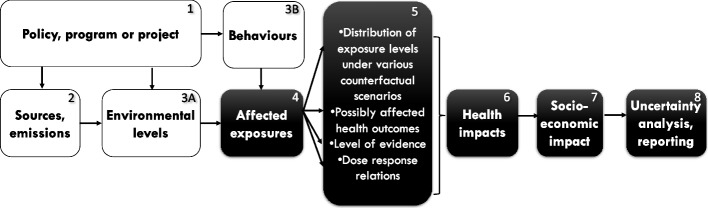
Overview of the main steps of risk assessment studies. The starting point of the study (or the counterfactual scenarios) can be formulated in terms of policy, program, project (1), environmental emissions (2), environmental level (3A), behavior (3B), human exposure (4)

These steps are depicted in Fig. 2. We first consider the simple case of a single environmental factor for which exposure levels are available or can be assessed (exposure being here understood in its strict meaning of the contact of an environmental factor with the human body). From the knowledge about exposure in the population under study and various other essential pieces of information detailed below, an estimation of the corresponding health impact will be provided, and generally compared to the impact under an alternative (or counterfactual) scenario (e.g., corresponding to the same population in which exposure is set to zero or another reference value, sometimes called TMREL, or Theoretical Minimum Risk Exposure Level, see Selection of the target scenario(s) – exposure levels). This health impact may be positive or negative, restricted to a specific symptom or disease, or consider several diseases or integrated measures of health such as Disability Adjusted Life Years (DALYs), which integrate years lost due to both death and disability.

Numerous variations around this basic scheme exist: one can start upstream of human exposure (box 4 in Fig. 2), for example from the environmental level of the factor (e.g., the amount of contamination of food by metals or the average atmospheric concentration of particulate matter [26], box 3A), further upstream, considering a source of potentially harmful or beneficial factors (e.g., a factory that emits several pollutants, the presence of green space [27]; box 2), or a behavior, possibly influencing exposures, such as burning incense, use of tanning cabins, use of electronic screens or cigarette smoking [28] (box 3B), or a policy/infrastructure or foreseen societal or environmental change (box 1), such as the ban of coal burning or of inefficient woodstoves in a specific area [29] or of flavored tobacco [30], the building of a school, or the temperature changes expected from climate change a few decades ahead [31], this policy, infrastructure or environmental or societal change being either real or hypothetical. In this latter case, some assessment (e.g., though modelling) of the variations of all the chemical, physical, psychosocial factors that may change as a result of the policy may be required, if it is not already available, e.g. as a result of a pre-existing environmental impact assessment study. One can also start downstream of exposure(s), in particular from a body dose or from the organ dose or the excreted level of an exposure biomarker. Depending on the starting point, specific data and modelling may be required, typically to translate the information about this starting point (say, a behavior or the presence of a factory) into an exposure metric that can be translated into a health risk, which will generally be the exposure metric for which reliable dose–response functions exist. Downstream of the health risk assessment, one may wish to also consider economic and other societal impacts.

### Compared situations

#### Principle of counterfactual comparisons

To estimate the impact of a given factor or policy, one needs to subtract the number of disease cases expected under the hypothesis that the policy is present or the factor(s) has a given distribution in the study population from the number of cases expected in the situation assuming that the policy is absent or different, or that the factor(s) has a different distribution. Either of these situations may correspond to reality as observed at a given time point. For this estimation to be relevant (i.e., for it to correspond to an estimation of the causal effect of the “change” implemented), the comparison has to be done in the same population and at the same time point. Otherwise, trends in the health outcome unrelated to the considered policy or factor may exist and bias the comparison between both situations. As an illustration, comparing the number of air pollution-related diseases in 10 years, after a road infrastructure has been built, with the situation today, in the absence of the infrastructure, would not distinguish the impact on health of changes in the traffic fleet in time from the impact of the infrastructure per se. In the terminology of causal inference theory, this corresponds to considering counterfactual situations. The reliance on counterfactual thinking is a basis for causal inference: the causal effect of a factor A corresponds to the difference between the situation in the presence of A and a (counterfactual) situation in which everything is similar with the exception that A has been removed [32, 33]. Note that the epidemiological concept of population attributable fraction developed before the application of counterfactual thinking to the health field, with the possible consequence that the counterfactual scenarios are not always explicit when estimating population attributable fractions.

The comparison can apply to the past, the current period, or to another period in the future. Therefore, the two main options are:

- *Counterfactual approach at the current period or in the past:* The number of disease cases (or healthy years lost because of specific diseases or another health metric) at the current time t_0_ in a hypothetical (“counterfactual”) world similar to the current world but for the fact that the factor is absent, or altered, or the policy has been implemented, is compared to the number of disease cases (or healthy years lost or another health metric) in the real world considered at the same time t_0_; t_0_ needs not to be to the time when the study is performed but can correspond to some time point in the past. We group the situations corresponding to t_0_ corresponding to current or past situations because in principle real data on exposures and health can be accessed for the default scenario. See e.g., examples in [34, 35].
- *Counterfactual approach with future scenarios:* Here, the number of disease cases (or healthy years lost because of specific diseases or another health metric) at a specific time t_1_ in the future in the study population in which the factor has been removed or altered, or the policy has been implemented, is compared to what would be observed at the same time assuming a specific reference scenario [29, 36, 37]. Time t_1_ might correspond to the time when the planned policy is expected to have been implemented, or some time later when a new stationary state is assumed to have been reached.

t_0_ and t_1_ are usually not time points but time periods over which impacts are summed; they may typically correspond to a one-year period, but may also correspond to a long duration, which may correspond to the life expectancy of the planned infrastructure, in which case the risk assessment may be repeated during each year of the study period to allow integration of the impact and possibly infrastructure or policy costs over this period. This is in particular relevant if the costs and benefits vary over time (the costs being possibly borne in the beginning and the positive impacts reaped after a longer duration). Such an integration allows to provide a relevant average of the yearly impacts and costs. An example is the assessment of the health, economic impact and cost of measures to limit air pollution over the 2017–2045 period, assuming that the measures have been implemented at the start of the study period [29].

The two options above may actually be combined, by providing estimates of the situation at time t_1_ in the future under various scenarios, together with an estimate providing to another earlier time t_0_ such as the current time. As an illustration, Martinez-Solanas et al. estimated the impact of non-optimal (both cold and warm) temperatures at the end of the twenty-first century under three greenhouse gas emission scenarios, but also at the historical period (1971–2005), allowing both to compare two possible futures with different levels of action against greenhouse gas emissions, as well as to compare the current situations with some possible futures [31].

Note that the first situation typically corresponds to attributable fraction calculations [7] done on the basis of the measure of association (e.g., a relative risk) estimated in an epidemiological study, using the exposure in the population from which the relative risk has been estimated. Also note that the observational study equivalent to the second situation corresponds to what is called the *difference-in-differences* approach [38]; in this observational approach, in order to estimate the impact of a real intervention, a community or group experiencing an intervention is compared to itself before the intervention, while another group that did not experience the intervention to correct this before-after comparison for temporal trends in the health outcome of interest).

A specificity of the second (“future”) option relates to the temporal evolutions of the study area. These possibly include changes in the demography (age structure and hence also possibly raw disease risk, as the incidence of most diseases varies with age), in specific disease risk factors besides the one in focus and also possibly regarding the dose response function, in particular for health outcomes very sensitive to societal changes, such as mortality. Such evolutions may be difficult to predict, in particular over periods of several decades or more. Illustrations include studies of long-term effects of ozone depletion [37] or of climate change, in which sociodemographic changes as well as societal adaptation to high temperatures [39] are expected.

#### Selection of the target scenario(s) – exposure levels

The scenarios correspond to the counterfactual situations that one wishes to compare to answer the study aim. One scenario will typically correspond to the *current* or *baseline* situation (if one is interested in the effect of a factor present today), or to the extension over time of the current situation, the so-called “business as usual” scenario, if the question asked pertains to a policy or infrastructure that would be implemented or built in the future. The alternative scenario(s) will correspond to the hypothetical situation in which the policy considered has been implemented (the policy being e.g., the construction of an infrastructure, a change in urban design, or a lowering of the pollution levels, if the study aims at quantifying the impact of a specific exposure). Of course, several counterfactual scenarios can be considered and compared (see examples in Table 1 or [29]).

**Table 1:** A series of 10 scenarios compared in a risk assessment study of the impact of atmospheric pollution (fine particulate matter, or PM_2.5_). From [40]

Scenario number	Scenario description	Scenario name	PM_2.5_ yearly level reduction
S1	Spatially homogeneous target value in the whole area	“WHO guideline”	Down to WHO yearly guideline (10 µg/m^3^ at the time of this publication)
S2		“No anthropogenic PM_2.5_ emissions”	Down to lowest nation-wide levels (4.9 µg/m^3^)^a^
S3		“Quiet neighborhood”	Down to lowest study area district levels (10th percentile of exposure)^b^
S4	Homogeneous PM_2.5_ decreases in the whole area	“-1 µg/m^3^”	Baseline^c^ -1 µg/m^3^
S5		“-2 µg/m^3^”	Baseline^c^ -2 µg/m^3^
S6	Targeted reduction in PM_2.5_-related mortality in the whole area^d^	“-1/3 of mortality”	Equivalent to decreasing homogeneously and sufficiently the baseline^c^ exposure to achieve the indicated health objective^e^
S7		“-1/2 of mortality”	
S8		“-2/3 of mortality”	
S9	2008/50/EU Directive^f^ “2020 target”	In the whole study area”	Baseline^c^ -15%
S10		Restricted to PM_2.5_ exposure hotspots”	Baseline^c^ -15%, only if baseline ≥ 90th centile of PM_2.5_ levels^g^

^a^Corresponding to the 5th percentile of PM_2.5_ concentration distribution among French rural towns

^b^The 10th percentile of PM_2.5_ exposure by Housing Block Regrouped for Statistical Information (IRIS) in the study area (corresponding to 10.3 and 12.4 µg/m^3^ in Grenoble and Lyon conurbations, respectively)

^c^Baseline corresponds to the PM_2.5_ exposure average for the 2015–2017 period, taken as a reference in the present study

^d^Mortality reduction targets expressed as a proportion of the non-accidental death cases attributable to PM_2.5_ exposure that can be prevented under the scenario S2: “No anthropogenic PM_2.5_ emissions”

^e^S6: -2.9 and -3.3 µg/m^3^ in Grenoble and Lyon conurbations, respectively; S7: -4.4 and -5.1 µg/m^3^; S8: -6.0 and -6.9 µg/m3

^f^Inspired by the 2008/50/EU Directive, which targets relative PM_2.5_ yearly average decreases to obtain by 2020. The decrease value depends on the exposure average for the last three years (2015–2017): -15% in the case of Grenoble and Lyon conurbations

^g^The 90th percentile corresponded to 16.0 and 17.4 µg/m^3^ in Grenoble and Lyon conurbations, respectively

An essential question here, for actions on one or several specific factors, relates to the targeted levels (or distribution) of the factor. In the case of a study aiming to quantify the current impact of a factor that has monotonic effects on all diseases considered, the counterfactual situation in which no one is exposed to the factor can be considered. This would correspond to the ban of the substance or the behavior considered, assuming that the substance is not persistent in the body or the environment, and that the compliance to the regulation is perfect. Other scenarios are however worth considering in specific situations; in particular, one may wish to consider levels strictly higher than zero as an alternative to the current situation if the factor corresponds to a substance persistent in the body or the environment (so that a ban would not lead to the immediate disappearance of the pollutant, as is the case for DDT [dichlorodiphenyltrichloroethane] or PCBs [polychlorinated biphenyls]), if it has both human and natural sources (as is the case for particulate matter, which are also emitted by volcanic eruptions) or if exposure to the factor does not have a monotonic association with disease occurrence (as is the case for outdoor temperature, exposure to sun radiations or level of essential elements in the body). The methodology of the Global burden of disease (GBD) project coordinated by IHME refers to a *Theoretical Minimum Risk Exposure Level* (TMREL) defined as the exposure level that “minimizes risk at the population level, or […] captures the maximum attributable burden” [14]. Alternatives exist, such as considering a *feasible minimum* (which may however require a specific approach in order to be rigorously identified), or specific existing guideline levels (e.g., WHO air quality guidelines [41]).

In the case of particulate matter (PM), studies in the early 2000s typically used WHO PM guideline value (then 10 µg/m^3^ for PM_2.5_) as the target, which then seemed a remote target – although this value did not correspond to a “no-effect level”, which has not been evidenced for PM_2.5_. Today, as exposures in many Western cities decreased close or below 10 µg/m^3^, lower reference values are often chosen, such as the 2021 WHO guideline value of 5 µg/m^3^, the lowest observed value, or the fifth percentile of the values observed across the cities of the country (which, in the case of PM, will lead to very different reference values in India and Canada, making between-country comparisons difficult), or an estimate of the levels that would be observed in the absence of anthropogenic source of PM. Since the “target” value has of course generally a very large impact on estimates, it is crucial for it to be explicitly quoted when summarizing the study results. Mueller et al. [36] give the example of a policy that would simultaneously target noise, air pollution, green space and heat exposure, as well as physical activity, taking for each exposure factor levels internationally recommended (in general by WHO) as counterfactual scenario (see Figure S1).

#### Considering environmental and societal side effects of policies

Ideally, the counterfactual scenarios should consider possible side effects and remote consequences of the considered intervention. For example, a risk assessment of a ban of bisphenol A (totally or for specific uses) could consider several counterfactual scenarios, one in which the consumer products containing bisphenol A are simply banned (possibly trying to consider the societal cost of such a ban if the products product a benefit to society), and others in which bisphenol A is replaced by other compounds, including some that also have possible adverse effects, such as bisphenol S. Studies on the expected impacts of climate change mitigation strategies, such as the limitation of fossil fuels use, may consider health effects due to the expected long-term improvement in climate, but also those related to changes in air pollution levels possibly resulting from the limitation of fossil fuels use, and also the possible consequences of increases in physical activity if this limitation of fossil fuels is expected to be followed by shifts from individual cars to other more active modes of transportation.

### Study area and population

The study area should be coherent with the policy or factor considered, trying to include the whole population likely to undergo positive or negative impacts from the factor or policy. Its choice should also take into account the entity (institution, community, administration, population…) able to take a decision regarding this policy. Choosing an area larger than that targeted by a policy makes sense, as it may allow to consider unplanned effects on the surrounding areas (for example, in the case of a policy planning to ban the most polluting vehicles from a city, an increase in the traffic of these vehicles in the surrounding cities), and to provide estimates specific to various sub-areas, which are also relevant because sometimes the exact area concerned with a possible policy is not always decided a priori – in which case the study may help making this decision. However, one should also keep in mind that considering a study area larger than that in which the policy will be implemented may entail possible dilution effects – i.e., the impact may appear lower than it is actually in the population targeted by the policy, if expressed on a multiplicative scale, that is, as a change in the proportion of deaths or DALYs in the area. When considering a policy decided at the city level, estimating the health impact in the city and possibly the surrounding ones is for example relevant; for a European policy, one may consider the whole Europe, or a (possibly random) group of regions if limiting the size of the study population limits bias, costs or uncertainties, which is not always the case for risk assessment studies contrarily to field studies.

In addition to factors related to health (see Assessment of disease frequency) and to exposure to the factor or policy considered (such as possibly fine-scale data on population density), it is usually relevant to collect information on population size, sociodemographic (documenting e.g., age and social-category distribution) and behavioral factors. Although seldom done in practice in the context of risk assessment studies, it is worth considering conducting a specific survey to document specific characteristics of the study population not available in administrative or health databases. For example, if the intervention may as a side effect impact physical activity (which has a non-linear relation to health), it is useful to document the distribution of physical activity in the population.

### Exposure (risk factors) assessment

#### Identification of exposures

In the simple case of a risk assessment study considering a single pre-identified factor A, before assessing the impact (Health impact), one has to:

- assess the expected exposure to factor A in the study population under the considered counterfactual scenarios (see Exposure assessment tools – general considerations to Reliance on exposure biomarkers);
- identify all health endpoints and possibly biological parameters H_1_, H_2_… H_i_ that can or could be influenced by A (see Identifying all health-related events possibly influenced by the considered exposures);
- assess the level of evidence regarding the possible effect of A on H_1_, of A on H_2_… H_i_ (see Estimating the strength of evidence about the effect of factors on health);
- assess the incidence of the health endpoints in the considered population, and the dose response function of all exposure-outcome pairs (A, H_1_), (A, H_2_)… (A, H_i_) (see Exposure-response functions).

As described in Fig. 2, the level of intervention can correspond to either the emission of factor A (e.g., what a chemical plant is allowed to emit on a yearly basis, box 1), its concentration in a milieu (e.g., air, water, food, box 3A), human exposure to A (referring, strictly speaking, to the contact of humans with the factor, integrating both the duration of the contact and the level of the compound, box 4); A may also correspond to a behavior (e.g., having sexual intercourse without using condoms, box 3B). We will here refer to all of these situations with the same simplifying terminology of exposure in the loose sense, keeping in mind that this differs from its strict definition given above, and that depending on the situation, one may have to do some modelling to translate the intervention into a metric compatible with the exposure response function to be used (see Exposure assessment tools – general considerations below).

If the starting point of the study is now a family of factors (e.g., endocrine disruptors, or environmental factors, as in the case of Environmental burden of disease assessments), then one may first have to list/identify which factors fall under this definition. This step can in practice be challenging and may require a specific technical study.

If the study aims at assessing the impact of a policy, one has to first analyze if and how the policy may translate in terms of environmental (understood in the broad sense) impacts – that is, identify which chemical, physical and social factors A_1_, A_2_… A_j_ may be influenced by the policy, and quantify the expected amplitude of the variations in these factors once the policy has been implemented. For example, if one aims at estimating the impact of banning a fraction of gasoline- and diesel-powered cars or trucks from an area, then one will generally have to rely on atmospheric dispersion models to estimate which changes in specific atmospheric pollutants will be induced by this ban, in addition to consider other consequences of the ban, e.g. related to physical activity. This actually corresponds to a study in itself (an environmental and social impact assessment), which may be already available, possibly because of legal requirements. One would then have to perform the three steps listed above (see end of Study area and population) for each of the factors A_1_, A_2_… A_j_, influenced by the policy, which may imply to assess and synthesize the evidence regarding a large number of exposure-outcome pairs (A_i_, H_j_).

The consideration of several risk factors has implications in the way the health impact is estimated, which are discussed in Consideration of multiple risks factors below.

#### Exposure assessment tools – general considerations

Whatever the starting point of the study (i.e., the targeted intervention or factor), the estimation of the health impact should ideally rely on some estimate of the exposure metric coherent with the dose–response function considered (see Exposure-response functions below), which should itself be chosen to minimize the uncertainties and bias in the final risk estimate. If, for example, the evaluated intervention corresponds to the closing of a plant emitting hazardous gases, one could attempt estimating the spatial distribution of the air concentration of the corresponding gases in the area surrounding the plant, averaged over a relevant time period, to convert this spatial distribution into an estimate of population exposure taking into account the spatial distribution of density of the target population (e.g., general population or any specific subgroup) in the study area, and possibly any relevant information on the time–space activity budget of the local populations, and then estimate the population risk from this estimated distribution of population exposure and a dose–response function chosen in coherence with this exposure metric. Similarly, if the intervention aims at changing a behavior such as having unprotected sexual intercourse, one would ideally need to obtain estimates of such behaviors before and after the hypothetical intervention, to provide an estimate of the incidence of a sexually transmitted disease.

All the tools of exposure science and etiological epidemiological studies can in principle be used to assess exposures, from environmental models to questionnaires, dosimeters and biomarkers, keeping in mind that they differ in terms of resolution, accuracy, cost, potential for bias…

As risk assessment studies are expected to provide an estimate relevant in a given population, representativeness of the group in which exposure is assessed with respect to this target population is a desired feature, contrarily to etiological epidemiological studies, for which representativeness is usually not required [42]. For this reason, “simpler” rather than more accurate and cumbersome approaches to exposure assessment than those used in etiological studies may be preferred, since the later, although more accurate at the individual level, may entail selection bias and thus have in some instances more limited validity at the population level. Consequently, environmental models or data, which may be developed without always requiring contact with the population, are very frequently used in risk assessment studies of environmental factors; we discuss below issues related to the spatial resolution of such models without entering into details of models development and validity not specific to risk assessment studies [43]; for behaviors, questionnaires may be used, while for many chemical exposures, data from biomarkers or exposure models may be preferred.

In addition to issues related to the validity of the exposure metric itself, errors may arise because of participation (selection) bias induced by the selection of the population for which this metric is available (e.g., answering a questionnaire or providing biospecimens to assess exposure biomarkers). Simply identifying a representative sampling base may be challenging in some areas if no relevant public database exists or if access cannot be granted; even if such a relevant sampling base exists, a participation rate of 100% cannot be expected, and refusing to participate can a priori be expected to be associated with sociodemographic characteristics and possibly with the exposure of interest. Tools such as reweighing approaches may be used to limit the impact of such selection bias on the estimated exposure distribution.

Again, whatever the approach used, the estimate of exposure provided should be coherent with the hazard identified and the dose response function to be used in the following steps (i.e., a dose–response function based on yearly exposure averages should not be combined with a weekly estimate of exposure).

#### Issues related to the assessment of environmental factors through questionnaires

Questionnaires may be used to assess behaviors, such as the frequency of use of specific modes of transportation (which would be relevant if one aims to quantify the impact of a policy entailing a shift in the share of specific modes of transportation), diet, smoking or physical activity and also psychosocial factors. Just like for environmental models, validation studies are particularly relevant to discuss and possibly quantify (see below, sensitivity analyses, Sensitivity and uncertainty analyses) any bias possibly induced by the questionnaire used, keeping in mind that the validity of questionnaires may be population-specific, as the validity of replies will depend on the social desirability of the considered behavior (for example, replies to questionnaires on alcohol consumption may be dependent on the social perception of alcohol consumption in the given cultural background), as well as evolve over time in a given society, limiting the validity of temporal comparisons.

When it comes to assessing exposure to specific chemical or physical factors, questionnaires may be very limited. Indeed, one is generally not aware of one’s own exposure to a chemical, in particular if exposure occurs through several routes (e.g., in the case of bisphenol A, PCBs, or specific pesticides), and cannot provide any quantitative estimate of her/his own exposure; additionally, one’s perception of exposures may be strongly influenced by social or psychological factors (e.g., one’s perception of the noxiousness of the factor, or the existence of a disease that one considers to be imputable to the considered exposure), which may bias the estimated impact (assuming that perception is not of interest by itself). As an illustration, a European study showed limited agreement of one’s self-declared exposure to traffic with an objective assessment of exposure, to extents that varied between countries [44]. The fact that questionnaires alone are typically limited to assess exposures to chemical and physical factors does not imply that they cannot be used in combination with other sources of information to provide a relevant exposure estimate (see Reliance on environmental models and surveys below). Moreover, questionnaires (including those relying on smartphone-based applications) are essential to assess behavioral factors such as dietary patterns, alcohol or tobacco consumption, physical exercise, transportation patterns, sexual activity… These often rely on data collected independently from the health impact assessment study, but there is no reason for investigators planning such a study not to envision an ad-hoc questionnaire survey.

#### Reliance on environmental models and surveys

In some areas, public authorities or other institutions developed “databases” regarding specific factors that may be a relevant source of information about the exposure of interest. These databases may be built from various sources and approaches. These include data on sources of specific hazards (e.g., maps on the location of pollution sources or on the chemical composition of cosmetics or other consumers’ products), environmental monitoring (e.g., databases on water contamination (typically based on measurements in the water distribution network, job-exposure matrices in an occupational setting or models), corresponding to boxes 2, 3A and 3B of Fig. 2 (the case of human biomonitoring is discussed specifically below). When available, databases on sources and environmental models and measurements have the advantage of being possibly representative of a specific milieu, in particular if they are based on environmental measurements or models, which can more easily be developed on a representative basis than measurements implying the participation of human subjects; they also have the advantage to rely on information often not known to the inhabitants of the study area (e.g., the level of contamination of the air or drinking water or food by specific compounds) and to possibly cover large spatial areas and extended temporal windows. They may provide source-specific information, which may be relevant if the health impact assessment study considers a possible intervention limited to a specific source of exposure; for example, atmospheric pollution dispersion models typically combine emissions from urban heating, traffic, industry… and may be used to predict environmental levels that would be observed assuming that emissions from one or several specific sources are decreased [29]. They possibly allow avoiding directly contacting individuals to collect information on their exposure, although in many situations it is actually relevant to combine such environmental models with questionnaire data: for example, questionnaires are essential to combine models or data on food contamination or dietary characteristics with individual information on food consumption patterns [45], models or measurements in drinking water benefit from information on water use (sources and amount of water drunk, frequency and temperature of baths and showers… [46], and atmospheric pollution models may be relevantly combined with individual information on time space activity, to integrate individual exposures across the places where each individual spends time [47, 48]. Indeed, environmental models typically provide an estimate of an environmental level and not an estimate of personal exposure in the strict meaning. Other issues related to their use is that such models do not exist for all environmental factors and areas and that their spatial resolution may be limited.

#### Environmental models—Issues related to spatial scale

**Fig. 3:**
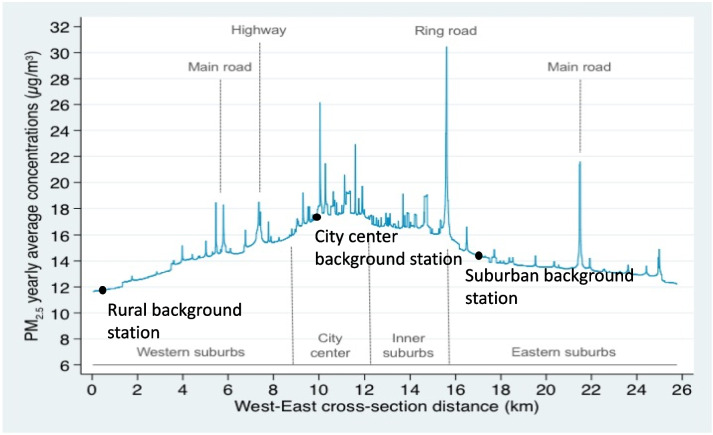
Cross-sectional variations of fine particulate matter (PM_2.5_) throughout the urban area of Lyon, as estimated from a fine-scale dispersion model, and typical locations of background permanent monitoring stations (black circles). Adapted from [40]

Indeed, the spatial resolutions of such models vary between models. In the case of atmospheric pollutants, early risk assessment studies typically relied on data from background monitoring stations, which cover a generally small fraction of territories and are sometimes distant by several kilometers one from another. In addition, background monitoring stations are by definition located in “background” sites, which are located far (typically, a couple hundred meters or more) from air pollution “hot spots” (see Fig. 3). On the one hand, relying on data from background monitoring stations may underestimate health impacts, as these background stations are not meant to represent people living or spending time close to air pollution hot spots such as industrial sources or high-traffic roads. On the other hand, stations located close to specific sources to monitor their activity are not meant to provide an estimate valid for a large area.

**Fig. 4:**
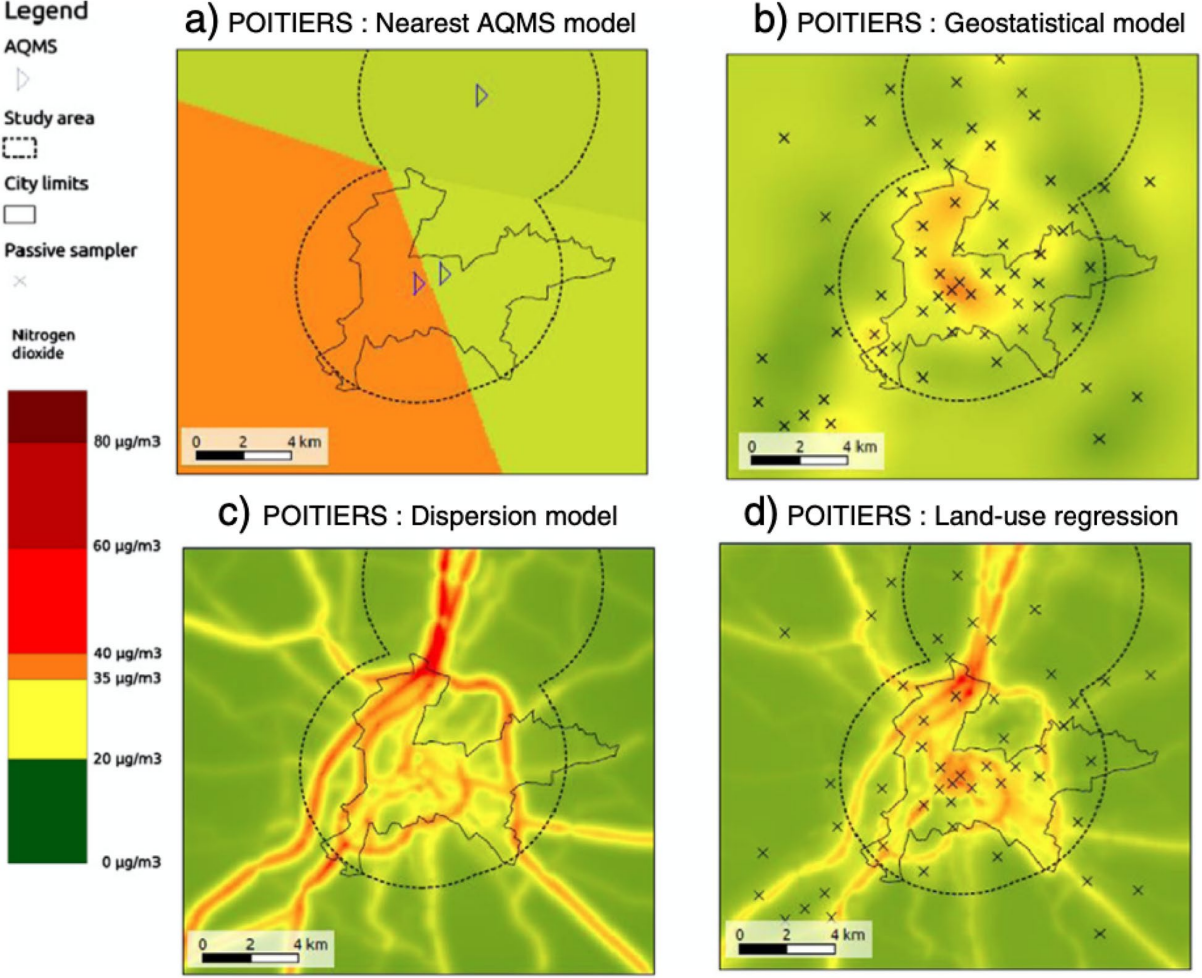
Spatial resolutions of various air pollution (nitrogen dioxide) exposure models developed in a middle size city. **a** Estimates based on permanent background monitoring stations; **b** geostatistical model relying on a fine-scale measurement campaign; **c** dispersion model taking into account emission and meteorological conditions; **d** Land-use regression model relying on the same measurement points as geostatistical model (**b**) [50]

In the last decades, models providing a finer-scale resolution, such as geostatistical models based on measurement campaigns in a large number of points, land-use regression or dispersion models were developed [49]. These have a much finer spatial resolution (Fig. 4). In a study in two French urban areas, assessing exposures from background monitoring stations entailed an underestimation of the mortality attributable to fine particulate matter by 10 to 20%, compared to fine-scale models with spatial resolutions taking into account variations in the pollutants’ concentration with a spatial scale of about 10 m [34]. Identifying the most relevant approach for risk assessment purposes is not straightforward. Even some fine-scale approaches may entail some error, as these may represent a “smoothed” version of the actual spatial contrasts in air pollution, and as smoothing typically entails a poor representation of the extreme values. These models with a very fine spatial resolution may be limited in terms of temporal resolution, which may be an issue for some health outcomes. Moreover and maybe counter-intuitively, relying on spatially very fine models may not be desirable in risk assessment studies in which there is no available data on the time–space activity of individuals. Indeed, if, in the absence of such individual time–space activity data, one has to assume that individuals are exposed at the concentration assessed at the location of their home address, then models that tend to smooth concentration over rather large areas may be more accurate for the purpose of risk assessment than models very fine spatially used ignoring the other places where individuals spend time.

#### Integrating environmental models with data on population density

As already mentioned, environmental models do not provide an estimate of the population exposure in the strict sense, if only because population density varies with space so that simply averaging the environmental levels over the study area, which gives the same weight to each location (and is equivalent to assume that the population is homogeneously distributed spatially across the study area) may poorly approximate population exposure. Getting closer to population exposure may imply to combine the estimated environmental level with data on population density (i.e., weighting concentrations with population density), which will allow considering the fact that the population is not evenly distributed in a given area. Kulhánová et al. [51] have illustrated these issues in a study of the lung cancer risk attributable to fine particulate matter exposure in France. Compared to a model that took into account PM_2.5_ exposure at a 2 km resolution and population density, a model ignoring the spatial distribution of homes within each *département* (geographical units of 200,000 to one million inhabitants) underestimated the population attributable fraction by about one third; when the variations in population sizes *between départements* was ignored, so that one assumed that everyone in the country was exposed to the median level observed at the country level, the estimated population attributable fraction was divided by 3.6, compared to the original one taking into account population density and fine-scale air pollution data (see Table 2). A large part of this bias was due to ignoring population density.

**Table 2:** Illustration of the influence of the spatial resolution of the exposure model and of the consideration of data on population density in health impact assessment studies (adapted from [51])

Hypothesis	PM_2.5_ exposure: 5th–50th–95th percentiles (µg/m^3^)	PAF (%) (95% CI)	Number of attributable lung cancer cases (95% CI)	Relative difference compared to main model (%)
**Approach 1: population-weighted PM**_**2.5**_** concentration (main model)**				
IRIS scale	8.3 – 13.8 – 21.8	3.6 (1.7–5.4)	1,466 (679–2,193)	–
***Sensitivity analyses***				
**Approach 2: population-weighted median PM**_**2.5**_** concentration**				
Department scale	9.7 – 13.8 – 19.1	3.6 (1.7–5.4)	1,471 (680–2,203)	0.4
Country scale	13.8 – 13.8 – 13.8	3.2 (1.5–4.9)	1,303 (598–1,965)	-11.1
**Approach 3: median PM**_**2.5**_** concentration without population weighing**				
Department scale	6.0 – 11.1 – 16.4	2.4 (1.1–3.6)	964 (445–1,446)	-34.2
Country scale	11.2 – 11.2 – 11.2	1.0 (0.5–1.6)	416 (190–631)	-71.6
**Approach 4: alternative RR of lung cancer (1.40 per 10 µg/m**^**3**^** increase in PM**_**2.5**_**, instead of 1.09)**				
Neighbourhood	8.3 – 13.8 – 21.8	12.9 (0.2–25.3)	5,232 (78–10,221)	256.8

The table gives the estimated population attributable fraction (PAF) of lung cancer cases attributable to fine particulate matter (PM_2.5_) exposure in France among subjects aged 30 years and more, for the year 2015 [51]

In approach 1 (main model), the PAF is estimated using a fine scale PM_2.5_ dispersion model (2 km grid) at the country level, averaged at the “IRIS” (neighborhood) scale and weighted by population density. In approach 2, exposure is smoothed by assuming that all IRIS of each département have the same PM_2.5_ concentration (corresponding to the median population-weighted value in each département), or that all départements in the country have the same PM_2.5_ concentration value (“country scale”). In approach 3, values also correspond to the median value at the département (respectively, country) levels, with the only difference compared to approach 2 that median value are estimated without weighting with population density

Approach 4 differs from approach 1 in that an alternative RR of 1.40 per 10 µg/m^3^ increase, obtained from a meta-analysis from ESCAPE project including 14 cohorts from eight European countries [52] is used, while a RR of 1.09 is used in model 1 [53]

*CI* Confidence interval, *PAF* Population attributable fraction, *RR* Relative risk

Note that at this step, the environmental levels and population density data can be combined with other spatially referenced data, such as information on sociodemographic characteristics, as a way to provide an estimate of how exposure distribution varies across these sociodemographic characteristics.

#### Reliance on personal dosimeters

Exposure assessment may also rely on personal sensors and dosimeters [54]. Generally, these have the advantages to provide an estimate of exposure that does not rely on detailed data on the sources of the factor, which are not always available (for many chemicals whose sources are not always monitored, such as benzene and other volatile compounds or pesticides), not on a modeling of the dispersion of the factor from its sources to the human environment, contrarily to the approaches discussed above in 2.4.4 to 2.4.6. Since dosimeters are carried by individuals, they efficiently allow taking into account the variability in exposure due to people moving between different environment [47]. They also allow to capture indoor levels of the factor of interest, which is of importance (for factors whose levels indoors and outdoors differ, such as ozone, benzene, radiations, temperature, noise…) given that people spend the vast majority of time indoors, at least in Northern countries. This increased “spatial” resolution compared to the above-mentioned environmental models (which typically capture outdoor levels, and generally at one location only if the time space activity of the population is not assessed) generally comes at the cost of possible limitations in terms of temporal resolution. In particular, it may be cumbersome to assess long-term exposure (which may be toxicologically relevant for specific outcomes) using personal dosimeters, which are typically carried over short-term (typically, from a day to a week) periods; these measurement periods may be repeated over time to improve the accuracy of the assessment as a proxy of long-term exposure [55], as discussed below for exposure biomarkers. Dosimeters are particularly relevant for media-specific exposures or factors, such as atmospheric pollutants including particulate matter [56, 57] or nitrogen oxides [47, 58], benzene [59, 60] and other volatile organic compounds [61], non-ionizing radiation such as ultra-violet [62] or ionizing radiation [63], temperature, noise [64]… Contrarily to environmental models, their use in the context of a health impact assessment study implies to recruit a population sample as representative of the target population as possible. Their use in risk assessment studies appears quite limited so far outside the occupational setting [61].

#### Reliance on exposure biomarkers

In the case of chemicals with multiple routes of exposure such as specific pesticides, which may be present in food, water and air, exposure biomarkers (the assessment of the compound or of its metabolite(s) in a tissue or fluid of the organism) may be a relevant approach. With the development of biomonitoring studies [65–67] and of cohorts collecting biospecimens [68], exposure biomarkers may be expected to be increasingly used in quantitative risk assessment studies related to chemicals.

Biomarkers study typically provide an estimate of the circulating or excreted level of the compound, which is not exposure in the strict sense but is related to it, while also depending on toxicokinetic factors, that typically vary between subjects [69]. Biomarkers integrate multiple routes of exposure, in that the level of a compound or its metabolites in a given body compartment will generally depend on the doses entering the body by ingestion, inhalation, dermal contact… (with many specificities according to the compound and tissue in which the metabolites are assessed). This may or may not be seen as an advantage, depending on the study aim, which may relate to exposure as a whole or to route-specific exposure (e.g., that due to food contamination). Considering these different routes is in principle possible via environmental models and measurements, but may be very cumbersome in terms of data collection and modeling work (consider for example a study on the impact of pesticides, which would have to estimate pesticide levels in water, possibly in the air, in food and to assess eating behaviors to try to reconstruct an individual’s exposure). A limitation of exposure biomarkers is related to the short half-life of many chemicals in the body, which implies that a spot biospecimen will generally be a poor proxy of long-term internal dose [70]. This is an issue for etiological studies, in which dose response functions assessed using a single biomarker in each subject are expected to suffer from bias towards the null [70]. This issue may also impact risk assessment studies. Indeed, even in the context of classical-type error, which may be hypothesized for biomarkers, although the population average exposure may not be biased when a spot measurement is done in each subject, the estimation of other features of the exposure distribution, such as variance and hence the estimation of specific exposure percentiles, is expected to be biased. Like questionnaire-based approaches, and generally to a larger extent, biomarker-based approach are dependent on individual’s participation and are therefore prone to selection biases; these may, again, be partly corrected if information on the factors associated with participation is available. Just like for all other approaches to assess exposures, behaviors and other health drivers, although rarely done, there is no reason beyond logistics and funding not to consider an ad-hoc biomonitoring survey as part of the HIA, on the contrary. We are not aware of specific quantitative evaluation of the bias associated with all the possible exposure assessment tools to a priori justify the choice of the exposure metric used in a given risk assessment study.

### Exposure–response functions

#### Identifying all health-related events possibly influenced by the considered exposures

For each factor (in the broad sense of chemical, physical, psychosocial, behavioral factor) primarily considered or identified at the previous stages as possibly influenced by the considered intervention, all health-related events that this factor might influence (the “health effects”, or hazards, a qualitative notion not to be mistaken with the quantitative health impacts) need to be identified. This identification should cover proximal and more remote effects, and positive (beneficial) as well as negative (detrimental) effects. For several environmental factors, the list of possible health effects may be long; for example, lead is a cause of neurological, nephrotoxic, cardiac, reproductive… effects, while particulate matter can affect cardiac, respiratory, metabolic and possibly reproductive and neurodevelopmental function [71]. Complex policies may entail numerous health consequences; for example, acting on traffic will affect air pollutants, but also noise, traffic accidents, greenhouse gas emissions, that may have long-term health effects (even if the corresponding impact may be limited, depending on the considered spatial scale). Even if the study does not provide a quantitative assessment of all the effects of a given exposure, identifying all of these effects is important. This identification of possible health effects should in principle rely on a systematic review that should encompass the human literature but also toxicology and possibly in vitro or in silico studies that may inform on mechanisms and point to specific health effects. Such an identification of all likely effects of the factor, change or intervention considered may rely on a recent well-conducted published review.

#### Estimating the strength of evidence about the effect of factors on health

The identification of each health effect possibly influenced by each considered factor should come with some assessment of the corresponding level of evidence. The assessment of the level of evidence evolved in the last decades from experts’ opinion to more formalized systematic reviews, possibly followed by meta-analyses and evidence integration approaches combining streams of evidence from various disciplines such as in silico data, in vivo and in vitro toxicology, environmental sciences, epidemiology… (see e.g., [72, 73] or the chapter 6 of [74] for a presentation of these approaches). Given the sometimes very large effort required by the implementation of such approaches, in particular for factors about which a vast literature exists, it is relevant to rely on existing assessments of the level of evidence, whenever a recent one with a transparent and relevant methodology is available. If not, the time and effort required for this step should not be underestimated, so that a review of the literature from all relevant disciplines, experts from these disciplines can be gathered to synthesize and weight the evidence and provide an assessment on a pre-specified grading scale (e.g., in terms of probability of causation). In case several factors are considered, then the number of exposure outcome pairs considered can be very large. An example of the assessment of the strength of evidence about endocrine disruptors is provided in Trasande et al. [75] and in Table 3.

**Table 3:** Estimated strength of evidence regarding the effect of endocrine disruptors on health

Exposure	Outcome	Strength of human evidence	Strength of toxicological evidence	Probability of causation, %
PBDEs	IQ loss and intellectual disability	Moderate-to-high	Strong	70–100
Organophosphate pesticides	IQ loss and intellectual disability	Moderate-to-high	Strong	70–100
DDE	Childhood obesity	Moderate	Moderate	40–69
DDE	Adult diabetes	Low	Moderate	20–39
Di-2-ethylhexylphthtalate	Adult obesity	Low	Strong	40–69
Di-2-ethylhexylphthtalate	Adult diabetes	Low	Strong	40–69
Bisphenol A	Childhood obesity	Very low-to-low	Strong	20–69
PBDEs	Testicular cancer	Very low-to-low	Weak	0–19
PBDEs	Cryptorchidism	Low	Strong	40–69
Benzyl and butyl-phthalates	Male infertility, resulting in increased assisted reproductive technology	Low	Strong	40–69
Phthalates	Low testosterone, resulting in increased early mortality	Low	Strong	40–69

The overall probability of causation (last column) was based on the toxicological and epidemiological evidence. From Trasande et al. [75] (extract)

#### Handling of the strength of evidence about the effect of environmental factors on health

In the past, a common practice was to only consider exposure-outcome pairs (A_i_, H_j_) for which the strength of the evidence regarding the effect of A_i_ on H_j_ was very strong or deemed causal. Another common option is to focus on a specific a priori chosen health outcome induced by the exposure, acknowledging that other effects are ignored; for example, many studies quantified the impact of tobacco smoke on lung cancer only, while other effects, e.g., on cardiovascular diseases, are certain.

The obvious consequence of these practices is to bias the estimated impact of the exposure or policy, generally in the direction of an underestimation (assuming that all associations go in the same direction, e.g., a negative effect of exposures on health). This is obvious for the second option above, but is also true for the first one. This is because in some cases, the discarded exposure-outcome associations will eventually turn out to correspond to very likely effects, as research continues, while the symmetrical situation of an effect deemed very likely or certain becoming unlikely as research unfolds is arguably much rarer in practice [76].

Possible alternatives to only considering very likely exposure–response pairs include:

- Considering all exposure-outcome pairs for which the estimated level of evidence is above a certain level (e.g., “likely association” or above, keeping in mind that diverse approaches are used to obtain these causality gradings and that various scales of evidence are used by various institutions), and estimating the corresponding impacts of these likely effects just like for the exposure-outcome pairs with a very high level of evidence. A special case consists in considering all exposure-outcome pairs for which there is at least “some evidence” of an association; in this case, there is potential for an overestimation of the overall impact (again, assuming that all associations go in the same direction);
- Performing sensitivity (additional) analyses considering exposure-outcome pairs with decreasing levels of evidence, and report the estimated impact of the exposure or policy considering only very likely effects, as well as the impact estimated considering also likely effects and suspected effects;
- Considering all exposure-outcome pairs for which the estimated level of evidence is above a certain level in the risk assessment and summing their impacts, weighing the sum by a weight increasing with the level of evidence of the corresponding effect, so that in the overall impact more weight is given to impacts from exposure-outcome pairs with a high level of evidence and less to the less likely ones.

Many studies more or less explicitly correspond to approach a). For example, the GBD methodology currently focuses on exposure-outcome pairs for which there is “convincing or probable evidence” [14]. An example of approach c), which is an intermediate one between the two first alternatives above, is a study of the cost of exposure to endocrine disruptors in the European Union [12, 75]. In this study, the health impact (and corresponding cost) attributable to exposure to each considered endocrine disruptors has been assessed using a Monte-Carlo approach in which, at each simulation run, the impact of a given exposure-outcome pair was possibly set to zero, according to the estimated probability of causation (with more likely effects being less often set to zero), and the overall impact was estimated averaging over many simulation runs and summed across exposure-outcome pairs. For example, in the case of a dose–response function of an exposure-outcome pair for which the strength of evidence had been rated to be about 50%, then the corresponding attributable cases were taken into consideration in only half of the simulation runs [75]. This approach seems relevant if a large number of factors is considered and if one assumes that the literature is not systematically biased. Indeed, if twenty factors are considered and the evidence is properly evaluated, then, assuming that the weight of evidence is estimated to be 50% for all factors, one may expect that eventually 10 of these factors turn out to really have an impact, so that counting half of the effect of the twenty factors may fall closer to the true impact (corresponding to that of ten factors) than if all twenty factors are ignored because the strength of evidence is not high enough. Note that the assumption regarding the fact that the literature is not biased can be debated for environmental factors, as for many factors, the weight of evidence typically tends to increase over time, rather than vary up or down randomly, and as a literature review of environmental alerts concluded that “false alarms” tended to be very rare in environmental health [76]. This would rather support not restricting the risk assessment to “certain” exposure-outcome associations, and also consider those with a lower level of evidence, possibly taking the level of evidence in the estimation as described above in c). Considering associations with less than certain level of evidence also allows to quantify the possible impact of suspected hazards, which is relevant to prioritize environmental factors for which research efforts should be dedicated [77]. In practice, the ability to implement the approaches depends on the availability of relevant data on exposures (which can be collected in the context of the risk assessment if not already available), exposure response functions (which may be very long and cumbersome to obtain if they are not already available) and baseline population health; this means that whatever the option chosen to handle the weight of evidence regarding each exposure-health outcome pair, the list of effectively considered pairs may be further restricted because of these issues related to data availability; this is expected to bias the health impact estimate (see Assessing the impact of policies versus the impact of exposures for further discussion). Transparency on all possibly affected outcomes is anyway warranted, even if not all of them can eventually be incorporated in the estimated overall impact.

#### Exposure–response functions

Exposure to chemical, physical and behavioral factors is now generally assessed on quantitative scales, and the expected impact of policies or plans on environmental and societal factor can often be translated in terms of quantitative variations in drivers of health. The ERF provides an estimate of the effect (positive or detrimental) associated with the exposure at each level (the terms of dose, concentration or exposure response function (DRF, CRF, ERF) or curve are here used synonymously). Issues related to the ERF relate to the validity of its assessment, including the slope and shape and to its applicability to the study population.

In the context of a risk assessment study, contrarily to the estimation of exposure which may in some cases be done ad hoc, it is generally not realistic to expect to generate from scratch a new ERF (whose estimation may require the follow-up of large populations over periods of years or decades) so that one has to rely on external ERFs. If no ERF is available, then one may either 1) try to derive an ERF from existing toxicological (animal) studies [78], if any or 2) perform a *qualitative* HIA. If several ERFs are available, then performing a meta-analysis of these ERFs to obtain a more robust one should be considered. This step may be done on the basis of the systematic review possibly performed to assess the level of evidence (see Estimating the strength of evidence about the effect of factors on health above).

**Fig. 5:**
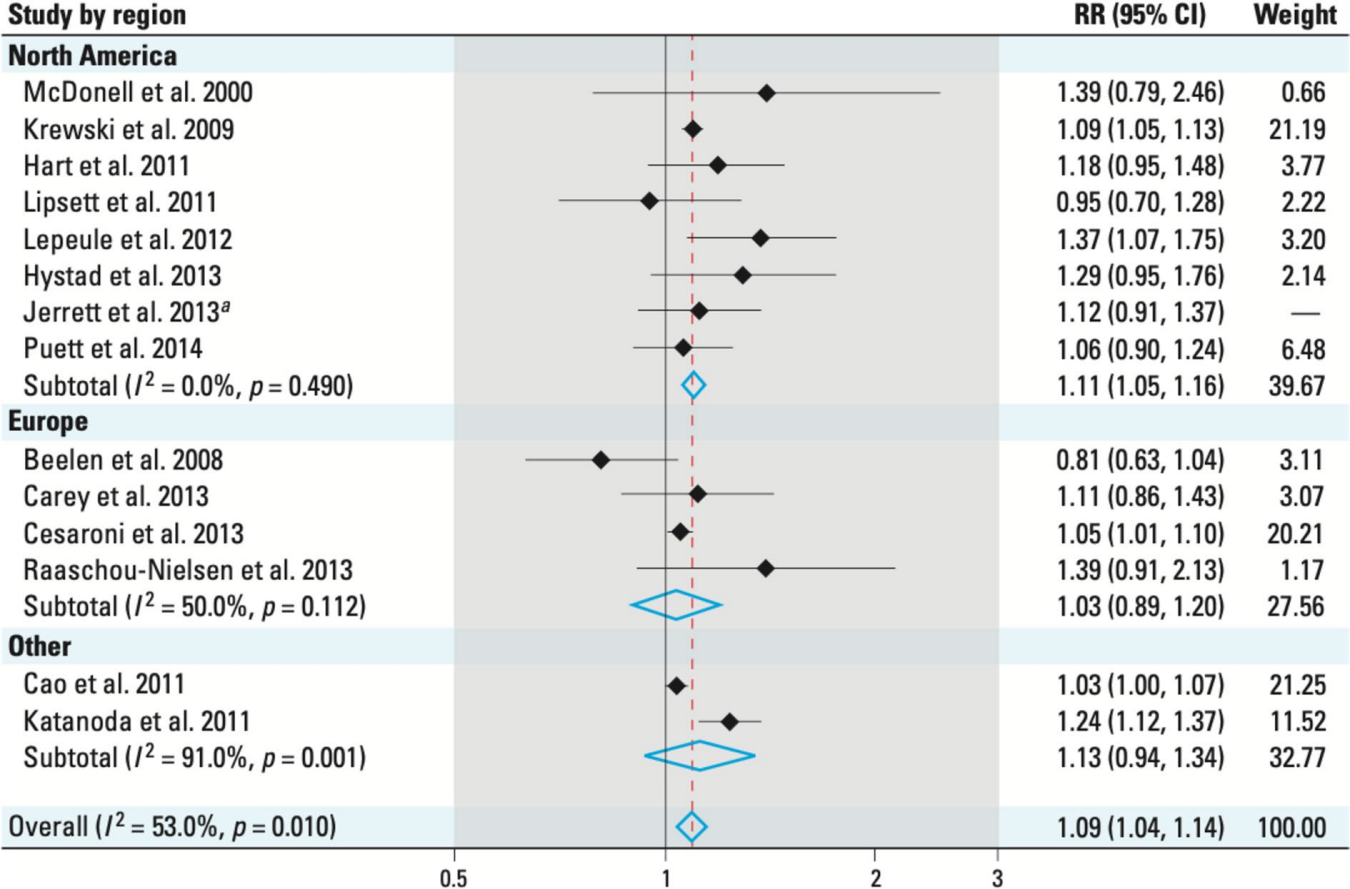
Meta-analysis of the relative-risk (RR) of lung cancer associated with PM_2.5_ exposure, by region [53]

We will here assume that some ERFs (or relative risks or any equivalent measure of association) are available. The choice of the exposure response function may have large influences, as illustrated by a review of air pollution HIAs illustrating that the estimated health impact of fine particulate matter in Europe varies by a ratio of two when switching between exposure–response functions based on two large studies [79] (see also Table 2). Three dimensions to consider are those of the sample size from which the ERF has been estimated, the potential for bias of the ERF, e.g., in relation to the adjustment factors considered, and that of its applicability to the study population. Researchers are typically left with the choice between an ERF estimated on a local (or nearby) population, which possibly relies on a small population (hence a large variance), or more global estimates, such as those from meta-analyses, which may be more robust (based on larger populations) but ignore possible differences in sensitivity between the local population and the rest of the world (and therefore are possibly biased). This can be seen as an illustration of the classical bias-variance trade-off. In an ideal situation in which many studies providing an ERF are available, one would characterize the potential for bias of each individual study (evidence evaluation, see e.g., chapter 5 in [74]) and then perform meta-analyses, in which the poor-quality studies would be discarded if they tend to produce different ERF estimates than the studies with better quality. The potential for heterogeneity in the ERF across populations and geographical areas should also be characterized (see Fig. 5), allowing to decide whether it appears more relevant to derive the ERF from a small group of studies in settings very similar to the setting of the HIA, or from a larger group of studies covering a wider diversity of settings.

It may be relevant to also consider other factors, such as the range of exposures in the studies from which the ERF is based, trying to base the ERF on studies with an exposure range similar to that of the population in which the risk assessment is conducted, and avoiding to extrapolate ERFs outside the exposure levels where the bulk of the original data lie. In the case of risk assessment studies focusing on factors of other nature, such as social or behavioral factors, for which the hypothesis of heterogeneity in sensitivity across large areas is more likely, the meta-analysis may not be the preferred option.

The concept underlying the exposure assessment in the study from which the exposure response function is based should be similar to that used in the risk assessment study. For example, if “exposure” in the study from which the exposure–response curve originates corresponds to lead environmental level, then it is generally not advised to rely on lead biomarkers (which assess the levels circulating in the body and not environmental levels) in the risk assessment study; the length of the exposure window should also be similar, as this length generally influences the estimated exposure variability. If both entities differ, then in some cases it may be possible to convert one into the other, using a formula derived from a study in which both entities have been assessed in the same population, or using toxicokinetic modelling. Contrarily to what is sometimes said, this requirement of a similarity of exposure concepts does not imply that the specific approaches used to assess exposures need to be identical. The measurement approaches can differ, provided one is not biased with respect to the other. For example, if the exposure considered is fine particulate matter and the exposure–response function stems from a cohort study in which exposure was assessed relying on permanent monitoring station, then a dispersion model could in principle be used to assess fine particulate matter levels in the risk assessment study. In this example, both the permanent monitoring stations and the dispersion models provide an estimate of the same entity (the environmental level), and since etiologic studies relying on permanent monitoring stations are not expected to be strongly biased compared to studies using environmental models with a finer scale such as a dispersion model (assuming that the concept of Berkson error [80] applies), the exposure–response function stemming from the study using monitoring stations is in expectation similar to the one that would have been obtained if a dispersion model had been used instead to assess exposure.

#### Non-linear exposure–response functions

The studied factor may have non-linear associations with the health outcome considered on a given (e.g., additive or multiplicative) scale. Note that such deviations from linearity are not always investigated in etiological studies (possibly for reasons related to limited statistical power). As an illustration, a 2001 review on the ERF of physical activity effects on mortality indicates that only 17 of the 44 studies conducted a test of linear trend [81]. A more recent and robust review does a meta-analysis on studies with larger population samples and finds a better fit for the curve y =—x ^0.25^, with steeper effect of moderate, as opposed to higher, physical activity on mortality [82].

Non-linear dose–response functions are all the more likely when the underlying mechanisms of action are complex and as the range of exposure values increases. Ignoring this non-linear relation can significantly impact the estimated risk [83], hence potentially misestimating the risks or benefits of the change, depending on the distribution of the exposure in the population.

**Fig. 6:**
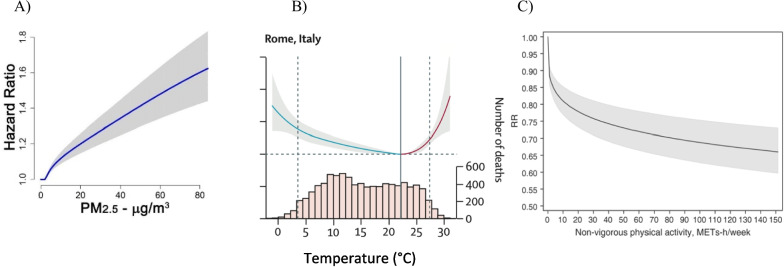
Illustration of non-linear exposure response functions: **A**) Fine particulate matter and mortality [85]; **B**) Temperature and mortality in Rome [86], **C**) Physical activity and cardiovascular events [87]. MET: Metabolic equivalents: RR: Relative risk

Non-linear ERFs have been exhibited for several risk factors. This is the case for temperature and air pollution effects on mortality in particular, and also effects of physical activity on health (Fig. 6); they may also be expected e.g., for endocrine disruptors [84].

Note that many risk assessment studies, at least from a certain period in the past, used to assume the existence of thresholds (hence, a non-linear dose–response) in the non-carcinogenic health effects of chemicals, and a lack of threshold of carcinogenic effects. There is to our knowledge no toxicological justification to such general claims. The “threshold” model can be seen as being related to the misconception that the NOAEL (no observed adverse effect level) estimated in some regulatory toxicology studies corresponds to a true “no effect” exposure level. In fact, a NOAEL generally corresponds to a level with an effect, whose size depends in particular on the number of animals used in the experiment aiming to estimate the NOAEL [88].

### Assessment of disease frequency

The ideal situation corresponds to that of an area where a register (or any other system allowing an exhaustive assessment of new disease cases on a representative basis) exists. Such registers exist in many countries for cancers but, outside Scandinavian countries, are rarer for other diseases. Just like for the case of the assessment of exposures, tools typically used in etiologic cohort studies may provide a relevant estimate of the disease frequency, with the same *caveat* as above, namely that etiologic studies are rarely representative of a given area, which would be a limitation if the disease frequency obtained in such a study is to be used in a risk assessment exercise. Alternatively, one can rely directly on estimates of the disease burden, such as those provided by the Global burden of disease project (https://www.healthdata.org/results/gbd_summaries/2019).

The disease frequency can correspond to different entities, generally related to incidence (the number of cases appearing during a given time period in a population) or prevalence (the number of cases present at a given time point, whatever the time when the disease started). In principle, incidence should be targeted. The entity used to assess disease frequency should be coherent with the measure of association (the exposure–response function) chosen. For example, a hazard rate stemming from a cohort study (or an incident case control study) assesses the change in the disease hazard (the strength of apparition of new cases) and needs to be combined with a measure of incidence and not prevalence.

### Health impact

#### Concepts of impact

The health impact (or risk) is the core estimate of a risk assessment study. It is a challenging notion, both from a conceptual and estimation perspective, not to mention issues related to the use of this expression with possibly different meanings across scientific and public health communities. When it comes to the human-derived risk assessment studies discussed here, the core product corresponds to notions close to the epidemiologic notion of attributable fraction. Following Greenland [8], we shall remind that this expression covers different concepts: the etiologic fraction, the excess fraction, the incidence density fraction, to which one shall add the expected (healthy) years of life lost.

The excess fraction corresponds to the proportion of cases that would not have become a case during the study period in the absence of exposure, while the etiologic fraction includes these excess cases as well as the cases due to exposure that would also have occurred during the study period in the absence of exposure, but at a later time point during this period. These two fractions can strongly differ because the etiologic fraction includes, in addition to the “excess cases”, the cases which would have happened also in the absence of exposure, but for which exposure made the incidence happen earlier than if the subject had not been exposed. These cases for which exposure simply made the case happen earlier in the study period may correspond to a large fraction of cases for complex diseases (as opposed to diseases with a simpler etiology such as infectious diseases), and their number increases with the duration of the study period. This is illustrated with the extreme example of a study of a factor increasing mortality or any other inevitable outcome: if the study period is very long, so that all members of the considered population are dead at the end of this period, then the excess fraction will become zero (because everyone eventually dies, even in the absence of the considered exposure)(see for example Fig. 2.6c in [89]), while the etiologic fraction may be non-null if the exposure does influence mortality [8]. For this reason, some advise not to use the excess fraction as a metrics, or the similar yearly number of avoided cases in a population [89]. Although this metric is indeed limited when it comes to estimating a meaningful health impact (that may be used to quantify an economic impact), when comparing the impact of various factors, it is possible that in many common situations the ranking of risk factors is preserved across metrics. In any case, it is of course crucial to only compare exposures in terms of impact assessed using exactly the same metric. Although in principle more relevant, the estimation of the etiologic fraction requires specific biological knowledge or hypotheses [8].

The incidence density fraction, defined as (ID_E+_-ID_E-_)/ID_E+_, where ID_E+_ (respectively, ID_E-_) is the incidence in the exposed (respectively, unexposed) group, has different interpretations, depending on whether one relies on instantaneous or average incidence densities [8]. Estimating attributable life-years (or healthy life-years) associated with the exposure or policy may appear as a relevant option for many public health purposes [8] and should be attempted. One reason is that this metric does not suffer from the above-mentioned limitation related to the fact that, since everyone eventually dies (the deaths are postponed, not avoided), the long-term gain expressed as a total number of deaths avoided from the reduction of exposure to a harmful environmental factor will appear much smaller than could be thought at first sight when considering the number of deaths avoided during a given year. The number of avoided life-years, by depending both on the number of deaths postponed each year and on the delay in their occurrence induced by the environmental change, takes into account the two dimensions of the problem. The same goes for the change in life expectancy [89]. Note that a given number of (healthy) life-years lost may translate into very different impacts on life expectancy, depending on how the life-years lost are distributed in the population (something that cannot be determined without strong assumptions). As an illustration, the UK committee on the medical effects of air pollution (COMEAP) concluded that anthropogenic fine particulate matter (PM_2.5_) at the level observed in 2008 in the UK was associated with an effect on mortality equivalent to nearly 29,000 deaths at typical ages in 2008, and that, depending on how this burden is spread in the whole population, this might correspond to impacts on life expectancy ranging from 6 months (if the air pollution effects was distributed across all deaths) to 11.5 years (if PM_2.5_ were only implied in 29,000 deaths) [89].

#### Estimation

The risk estimation relies on a more or less sophisticated combination of the exposure distribution under each counterfactual scenario with the ERF and with an estimate of the baseline disease frequency in the considered population, whether explicitly or hidden in the externally available disease burden. This estimation is repeated under each of counterfactual scenario, and the risk difference between the targeted and “baseline” scenario is computed. In practice, several ways to estimate the risk are used, which are not all strictly valid in theory. The main types of approaches correspond to:

- *PAF-based formulas:* An analytical (formula-based) estimation of a “population attributable fraction” associated with the exposure, multiplied either by the incidence of the disease or by an externally available estimate of the disease burden in the population (i.e., the impact of the disease in the considered population irrespective of all its possible causes, typically, expressed in DALYs);
- *Person-years method:* A simulation of the whole population in which new disease cases occur each year during the course of the study period under various counterfactual scenarios, from which attributable cases, differences in life expectancy, DALYs and other specific measures of risk can be estimated. This approach has the advantage of allowing to take into account the dynamics between death rates, population size and age structure [89].

Note that alternative approaches exist, such as compartmental models (generally, but not exclusively, used for infectious diseases) or approaches based on individual, as opposed to population-based modeling, such as microsimulations (see e.g. Mueller et al. [90] for a review). Compartmental models assume that subjects switch between mutually exclusive states (e.g., susceptible, infected, recovered, dead, for an infectious disease) and model the trajectory of each individual from the population across these states via deterministic or probabilistic approaches. They are particularly relevant to model the impact of interventions that may influence infectious diseases, and will not be detailed here (see [91] for an example). The lifetable (or person-years) approach mentioned below can in a way be seen as a particular example of compartmental models.

As already mentioned, one general issue relates to the consistency between the various metrics used; for example, the data on baseline disease frequency need to correspond to an estimate of disease incidence if the exposure–response function is derived from an etiologic study assessing the occurrence of new disease cases, such as a cohort or incident case-controls study.

The “PAF-based formula” approach can be illustrated taking the simple example in which a binary exposure level is changed from 1 to 0 (e.g., all smokers stop smoking) in the counterfactual situation and in which the overall burden associated with the disease, assumed to correspond to a dichotomous outcome (with/without disease) is available. The health impact is generally estimated in two steps, the first one corresponding to the estimation of the PAF, which, in a situation without confounding, is defined as:

1 $$PAF= \frac{P\left(Y=1\right)-P(Y=1 /\mathrm{ X}=0)}{P(Y=1)}$$

Where Y corresponds to the disease, P(Y = 1) is the proportion of subjects developing the disease in the study period and X is the exposure of interest, with X = 0 corresponding to non-exposed subjects. Equivalently, the PAF can be defined as:

2 $$PAF= \frac{P\left(X=1\right)\times ({R}_{e}-{R}_{u})}{P(Y=1)}$$

Were R_e_ and R_u_ are the risks of disease in the exposed and unexposed subgroups, respectively.

Note that the assumption about the lack of confounding can be conveniently ignored by relying on the structural causal modeling framework [92] and the *do* operator:

3 $$PAF= \frac{P\left(Y=1\right)-P(Y=1 / do({\text{X}}=0))}{P(Y=1)}$$

where do(X = 0) refers to a situation in which X is set to 0 through a minimally invasive intervention (in the terminology of Pearl [92]), all other variables possibly influencing X remaining constant; of course, the “reference” value X = 0 can be replaced by any other value or distribution, in the case of an exposure with more than two categories.

Coming back to the situation in which X is binary, the PAF is generally estimated relying on Levin’s formula, which can be derived from the previous ones:

4 $$PAF= \frac{P\times (RR-1)}{1+P\times (RR-1)}$$

Where P is the prevalence of exposure in the population, or P(X = 1), RR the relative risk associated with exposure (assumed here to be dichotomous). (Note that Rockhill et al. [6] explain that this formula is not valid in the context of confounding. This is true when one applies the formula (4) in the study population from which the RR is estimated but not, as in a risk assessment exercise, if one uses (4) to estimate the attributable fraction in a given population using a RR assumed to be unbiased estimated from another population.

The health impact is then estimated by combining the estimated PAF with the burden of the considered disease, BD, in the study population, generally available or approximated from external sources (or estimated via an ad-hoc study):

5 $$Health \,Impact=PAF\times BD$$

The unit of BD (e.g., deaths, DALYs, etc.) defines the unit of measure of the health impact.

In the case of a categorical assessment of exposure, the health impact is estimated as above in each exposure category, after which the overall impact is estimated by summing over all exposure levels. If exposure is continuous, formula (1) above is generalized by integrating the PAF over all exposure levels as shown here:

6 $$PAF=\frac{{\int }_{x=0}^{m}RR\left(x\right)\times {P}_{S1}(x)dx- {\int }_{x=0}^{m}RR\left(x\right)\times {P}_{S2}(x)dx}{{\int }_{x=0}^{m}RR\left(x\right)\times {P}_{S1}(x)dx}$$

Where m is the maximal exposure level, P_S1_ is the observed distribution of exposure level (or baseline scenario), P_S2_ the distribution of exposure under the counterfactual hypothesis (which may correspond to a uniform distribution with all subjects at zero if zero is the targeted level) and RR the exposure–response function, with RR(x) providing the relative risk when exposure is x.

If the health parameter Y is continuous (e.g., blood pressure, birth weight…), then the impact of X on the average value of Y can be estimated as:

7 $$Health \,Impact={\int }_{x=0}^{m}\beta \left(x\right)\times {P}_{S1}(x)dx- {\int }_{x=0}^{m}\beta \left(x\right)\times {P}_{S2}\left(x\right)dx={\int }_{x=0}^{m}\beta \left(x\right)\times \left[{P}_{S1}(x)-{P}_{S2}(x)\right]dx$$

Where β corresponds to the exposure–response function describing the average value of the outcome Y as a function of the outcome. In the case of a binary exposure with prevalence P, the right-hand side of this formula simplifies to β x P. This value can be multiplied by the population size if one wants to express the impact in terms of units of Y (e.g., IQ points) due to the exposure in the population as a whole.

The person-years approach consists in simulating the cohorts corresponding to each of the considered counterfactual scenarios throughout the study period, with new disease cases appearing each year. It has several key advantages over the formula-based approach, including: 1) to make all assumptions more explicit; 2) to avoid issues related to the estimation of the expected number of cases [7, 93], since the number of subjects still at risk in each cohort is explicitly estimated; 3) to be more flexible in coping with various exposures simultaneously, assuming various correlation structures between them, with scenarios implying gradual changes in exposure over time, considering sociodemographic changes in the study population, without having to work out an analytical solution. The cost of this approach is that it is generally much more complex to implement and compute.

The estimation needs to be repeated for the other health outcomes identified at the hazard identification step (Identification of exposures above) as being possibly influenced by the considered factor. It can also be repeated for other factors, as we now discuss.

#### Consideration of multiple risks factors

If several factors are considered (e.g., because one is interested in a prespecified set of exposures, or because the policy evaluated is expected to influence several physical, chemical, psychosocial factors), the estimation needs to be repeated for each of these factors, at least those for which the level of evidence regarding effects on one health outcome are above the selected level, if any (see Handling of the strength of evidence about the effect of environmental factors on health). A central issue here relates to the situation in which two or more of these factors can influence the same health outcome. Indeed, care is required to acknowledge the fact that the fraction of cases of a specific disease attributable to different risk factors can generally not be summed. This is a consequence of the multiplicative nature of risk and of the multifactorial nature of most diseases [94]; moreover, care is needed to consider possible relations, and in particular correlation, effect measure modifications or mediation between risk factors.

Again, the PAF-based formula and the person-years method can be used when considering several factors influencing the same health outcome, with the latter being more flexible. Regarding the former approach, if population attributable fractions have been estimated for each of the R risk factors influencing the considered outcome, and under some hypotheses (see below), then these can be aggregated with formula (6):

8 $$PAF= 1-\prod\nolimits_{r=1}^{R}{(1-PAF}_{r})$$

Where PAF_r_ is the population attributable risk fraction associated with risk factor r estimated independently from the other risk factors. This approach makes strong assumption: that all risk factors act independently (in particular, that no effect of one factor is to some extent mediated by another factor, or modified by another factor) and are not correlated. Note that this formula is identical to that used in toxicology for the so-called case of *independent action* [95]. Under these assumptions, if two independent factors have attributable fraction of the cases of 40 and 50% respectively, then their joint action corresponds to an attributable fraction of 70% (40% plus 50% of the remaining 60%).

Some of these assumptions may not hold in real situations.

A first issue relates to the situation in which exposures to the considered factors (say, x_1_ and x_2_) are correlated. Formula (8) assumes that the fraction of cases attributable to B is the same in the whole population and in the population from which factor A has been removed (independent action), which does not hold if A and B are correlated because then the prevalence of exposure to B is not the same in the two populations. From this it appears that information on the relations between exposures to the considered factors (here, x_1_ and x_2_) in the study population is required to estimate the fraction of cases attributable to x_1_ and x_2_. Specifically, their joint distribution P(x_1_, x_2_) needs to be considered in the PAF estimation, as described in Ezzati et al. [96]. Formula (6) can be adapted by replacing each integral by:

9 $$\underset{{x}_{1}=0}{\overset{{m}_{1}}{\int }}\underset{{x}_{2}=0}{\overset{{m}_{2}}{\int }}{RR}_{1}\left({x}_{1}\right){RR}_{2}\left({x}_{2}\right)P\left({x}_{1},{x}_{2}\right){dx}_{1}{dx}_{2}$$

this implies of course that information on the joint (and not only marginal) distribution of all relevant factors is available or collected in the population in which the risk assessment is conducted. Biomonitoring and exposome studies, provided they assessed multiple exposures simultaneously in the same participants, allow providing such a joint distribution [97]. Equation (9) assumes that the risk for a given combination (x_1_, x_2_) of exposures X_1_ and X_2_ is the product of the relative risks associated with X_1_ and X_2_, corresponding to a hypothesis of lack of effect measure modification of X_1_ by X_2_.

If now there is evidence of effect measure modification (sometimes termed interaction) between risk factors then in principle RR_1_(x_1_).RR_2_(x_2_) in Eq. (9) should be replaced by RR(x_1_,x_2_), that is the relative risk function describing the joint effect of x_1_ and x_2_, which can incorporate a different relative risk associated with x_1_ at each given value of x_2_. To our knowledge, there are currently few examples of risk factors and outcomes for which this function is accurately characterized and available.

Another option to handle it is to consider different ERFs in different population strata; again, one needs information on the joint distribution of all relevant factors, as well as stratum-specific relative risks.

**Fig. 7:**
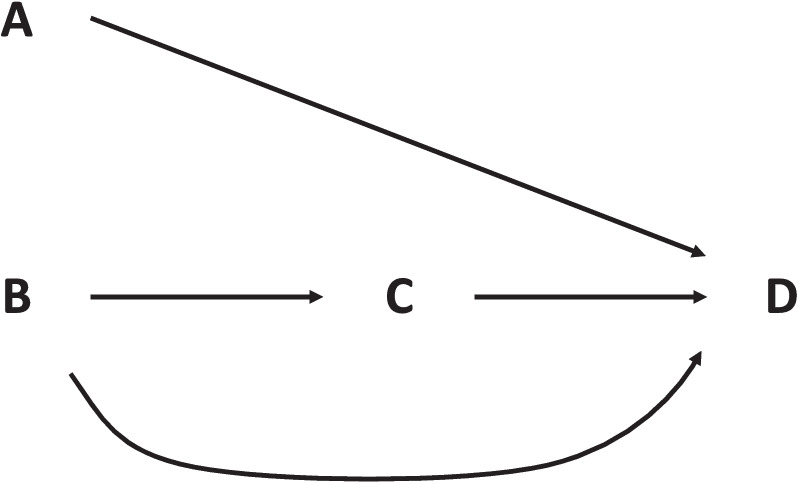
Causal diagram summarizing the causal relations between hypothetical risk factors (**A**, **B** and **C**) and a disease **D**. Here, **A** and **B** are assumed to independently affect the probability of disease, while a part of the effect of **B** on **D** is mediated by **C**

Another (non-exclusive) situation is that of mediation effects [98]. Consider the case of a disease D (say, lung cancer), influenced by several risk factors including active smoking (A) and green space exposure (B), the effect of which being partly mediated by changes in air pollution levels (C). Figure 7 provides a possible model of the assumed relations between A, B, C and D. Let us assume that one is interested in estimating the overall impact of factors A, B, C on D, that is, the number (or the fraction) of cases of disease D that would be avoided if A, B, and C all had “optimal” levels. The estimated impacts of B (improving green space exposure) and C (getting rid of air pollution) cannot be considered as being independent because a part of the effect of B is included in the effect of C (a mediation issue). Estimates of the share of the effect (in the sense of measure of association) of B on D that is not mediated by C (but that may be mediated by other factors not considered here, such as an increase in physical activity), termed the natural direct effect, can be provided by mediation analysis techniques [98]. This natural direct effect of B on the disease risk is by construction independent of the effect of C on disease risk, so that the corresponding attributable fractions can then be estimated and combined using formula (8) above. In the Global Burden Disease methodology, for each pair of risk factors that share an outcome, the fraction of the risk mediated through the other factor is estimated using mediation analysis techniques, if the relevant studies (in which B, C, D are altogether estimated) are available. A concern here (besides the usual assumptions required by mediation analysis [98]) relates to the transposability from one area to the other of such mediation analyses; for example, the change in air pollution level following a change in green space surface may be influenced by the local share of traffic-related air pollution among the total of the emissions of air pollutants from all sources, which may vary across areas.

In the case of a continuous health outcome, one option to estimate the joint impact of several factors, which assumes a lack of synergy (or of departure from additivity) is to sum the average changes in the outcome attributed to each exposure to obtain an estimate of the impact of the combined exposure.

#### Cessation lag

**Fig. 8:**
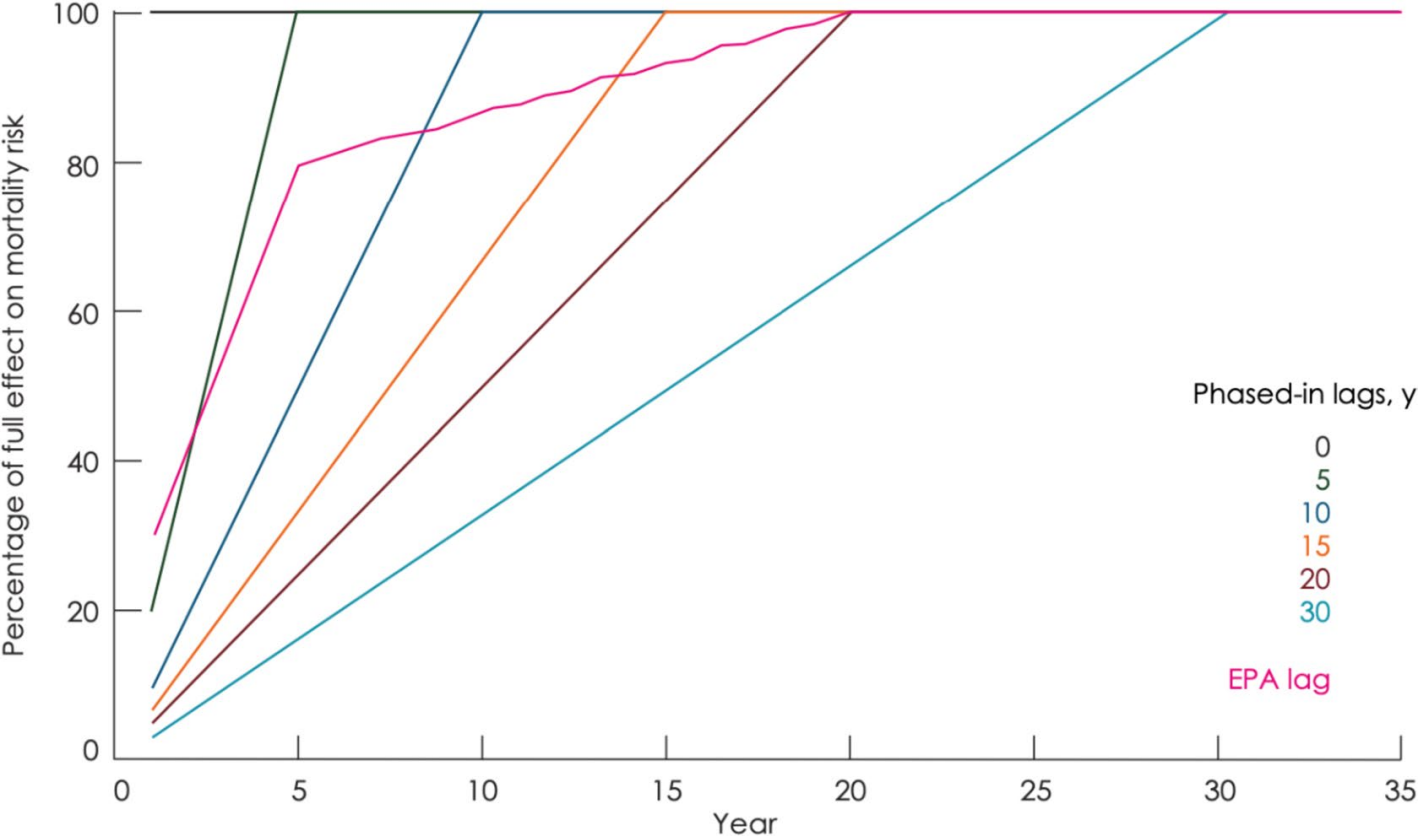
Illustration of possible cessation lags (in years) considered in the estimation of the impact of fine particulate matter exposure on mortality [89]. The first year of the intervention implementation is designated as year one

The cessation lag can be defined as the time lag between the implementation of the considered intervention and the consequent change in hazard rate. It is meant to take into account the fact that for most (chronic) clinical endpoints, the effect of changes in (external) risk factors does not manifest fully immediately. The COMEAP study of particulate matter impacts on mortality [89] provides an illustration of the impact of various cessation lags (see also Fig. 8). Such a cessation lag can be implemented in studies relying on person-year approaches. Whether a cessation lag needs to be considered depends on the availability of knowledge about when a change in exposure will start influencing the considered health outcomes, as well as on the question asked: if for example one is interested in knowing how health is likely to vary in the short and mid-term if one managed to implement a given intervention now (or in a near future), then considering a cessation lag is relevant; if the question of interest is the more theoretical one of quantifying how much better the population health would be today if a given exposure or set of exposure was absent (or if a specific intervention had been implemented a long time ago), then cessation lags might be ignored.

### Socio-economic impact of attributable health effects

One may wish to go beyond the health impact estimates. Economic analysis can further inform choices between different project or policy alternatives considering dimensions beyond health. Such an analysis can be limited to quantifying the costs of implementation of the considered scenarios or policies (e.g., those required to induce a reduction of exposure to a specific factor), and relate these costs to the health benefits expressed in non-monetary terms, such as DALYs (corresponding to a cost–benefit analysis). Beyond this, cost–benefit analyses have the advantage of allowing to compare the costs and monetized health and non-health benefits. Such an analysis is more complete and may be more meaningful for decision-makers than one ignoring the costs of implementation of each of the considered alternatives.

The benefits include the monetization of health benefits, which takes into account both tangible and intangible costs. The tangible costs refer both to direct costs, in particular the costs to the health system (costs of specific treatments for each pathology: drugs, consultation, hospitalization, etc.) and indirect costs linked to absenteeism and the resulting loss of productivity. The intangible costs refer to the inclusion in the economic analysis of the loss of well-being due to anxiety, discomfort, sadness or the restriction of leisure or domestic activities due. These are therefore non-market costs for which it is necessary to reveal their value through survey methods or analysis of the behavior implicitly attributed to them. For example, Ready et al. [99] and Chilton et al. [100] valued the willingness to pay to avoid an air pollution-related morbidity episode. Among these intangible costs, the economic valuation of mortality is a delicate step from an ethical point of view in the absence of consensual values. It is generally based on the monetary valuation of a statistical life or a year of life lost. In recent years, the most popular approach in the economic literature to determining the statistical value of a human life is the willingness-to-pay approach. The statistical value of a human life is thus approximated by the amount of money a society is willing to pay to reduce the risk exposure of each of its members. This literature shows that the statistical value of a human life depends on characteristics such as age at death, time between exposure and death (i.e. latency), and nature of the underlying risk [101, 102]. Empirical assessments have provided a range of values generally between €0.7 and €10 million per human life. The other approach used is that of revealed preferences, which is based on observed behavior: for example, the difference in wages between two branches of economic activity with different mortality risks.

The cost–benefit analysis, beyond the health benefits directly generated by the project or policy, can also integrate co-benefits not directly related to health. For example, Bouscasse et al. [29] considered the following co-benefits of measures to reduce fine particle pollution: the reduction of noise, the development of active mobility on health, which lead to health co-benefits, but also the reduction of greenhouse gas emissions, which includes benefits not related to health.

With regard to the evaluation of costs, it is necessary to define the scope: is it the cost of implementing the policy for the public authorities? Should the impact on household expenditure also be taken into account? This may be important, for example, in the case of measures aimed at reducing air pollution through actions on heating or transport, for which individuals carry a part of the cost. The assessment may also seek to quantify the impact on employment, for example, or on imports or exports of goods and equipment.

Finally, the time dimension is important in the implementation of a cost–benefit analysis. While costs usually occur at the beginning of the period (implementation of actions or policies, investments), benefits tend to occur later. Indeed, health benefits are generated over time depending i) on the speed of implementation of actions and of the progressiveness of the reduction of exposures, and ii) on the fact that health benefits may not be immediate following the reduction of this exposure (cessation lag).

The time lag between costs and benefits has another consequence in a cost–benefit analysis. We do not give the same value to €1 to be paid today or in several years, because of what economists call the preference for the present. This is introduced into cost–benefit analysis through a *discount rate*, which gives a present value to monetary flows in the future. The higher the discount rate used by the public authority or by the stakeholders, the lower the present value of benefits that occur later.

### Sensitivity and uncertainty analyses

#### Sources of uncertainty in quantitative risk assessment studies

Uncertainties exist at each step of risk assessment. For example, there may be uncertainties in the health outcomes influenced by the considered factor or policy, in the level of evidence relating the factor or policy with a given health outcome, in the corresponding dose–response function, in exposure distribution… We will here distinguish uncertainties due to the variability of the quantitative parameters considered in a risk assessment study (typically, in the dose–response function, but also possibly in parameters of higher dimension, such as the distribution of an exposure in the population) from more systemic uncertainties related to the model choice (sometimes termed *systemic* or *epistemic uncertainties*, e.g. related to the assumption that the risk factor(s) considered only influences a specific health outcome, possibly disregarding health effects not identified yet or for which a dose–response function is not available) [103]. A typology of sources of uncertainty in burden of diseases studies is presented in Knoll et al. [104]. We will focus here on uncertainty due to the variability in parameters, and touch upon systemic uncertainty in Sect. "Sensitivity and uncertainty analyses" below.

The consideration of the uncertainty related to variability typically implies to obtain an estimate of the uncertainties occurring at each step of the study, in order to combine these uncertainties (uncertainty propagation, or error propagation) and try providing an estimate of the resulting uncertainty on the overall study results.

#### Estimating the impact of uncertainties

In the simple case of a single source of uncertainty, the translation of this uncertainty on the overall results is in principle relatively straightforward; for example, if one only considers uncertainty on a dose response function expressed using a relative risk and (simplistically) assumes that this uncertainty is conveyed by the confidence interval of the relative risk, then the estimation of the health impact can be repeated using the limits of the confidence interval instead of the point estimate of the relative risk (of course, the confidence interval usually only conveys uncertainties related to the population sampling, more specifically to random error related to sampling.

However, there are multiple sources of uncertainty beyond those related to sampling error. Indeed, in the classical view of epidemiology (developed for measures of association), the uncertainty due to variability can be seen as having a random and a systemic component; only the former is easily estimated, while the estimation of bias requires quantitative bias assessment methods [105] that are seldom applied. In particular, sources of uncertainty related to exposure measurement error, to the assessment of disease frequency, possibly confounding bias or uncertainties in the shape of the exposure response function and the existence and shape of cessation lag, are not conveyed by the confidence interval of the relative risk and are worth considering – but rarely taken into account.

If one tries to simultaneously take into account several sources of uncertainty, then more complex approaches are required to propagate the uncertainty down to the impact estimate. Although analytical approaches (such as the delta-method) may be applicable in relatively simple situations, one more general approach corresponds to Monte-Carlo simulations. Monte-Carlo simulations rely on the principle of repeating a large number of times the health impact estimation, letting all underlying parameters (relative risks, exposure distribution, possibly the number of factors influencing the health outcome and the outcomes that they influence, if there are uncertainties at this level…) vary across realistic values [106, 107]. They allow providing an estimate of the distribution of the possible values of the health impact. This requires knowledge or assumptions on the likely distribution of each parameter considered in the study. If such an approach is implemented, then authors will be able to report a distribution of the most likely value of the health impact or cost. The results will for example be conveyed in a way such as: “Taken all identified sources of uncertainty into account, there is a 90% probability that the number of deaths attributable to factor A in the area is above 10, a 50% chance that it is above 30 and a 10% chance that it is above 50 cases” (with of course specific explanations for a non-scientific audience). Alternative approaches to Monte-Carlo simulations also exist, in particular in the context of Bayesian modeling [108]. Provided relevant data are available, this framework can in principle accommodate both the uncertainty related to variability, but also systemic uncertainty [109].

In the absence of formal consideration of the systemic uncertainty in the uncertainty analysis, it remains essential for the investigators to state their model’s assumptions and limitations, including in particular the impacts related to specific risk factors or health outcomes that could not be taken into account in the quantitative assessment (see also 3.3 below).

## Conclusion

Quantitative assessment studies are at the interplay between scientific, policy and legal issues; contrarily to what the deceptively simple epidemiological concept and formula of the “population attributable fraction” may let think [7, 8], their implementation and interpretation is very challenging.

We have reviewed some of the possible approaches and issues at each step of risk assessment studies. We have made the choice not to discuss the steps of problem framing, study reporting, and issues related to population participation, which are presented elsewhere [19, 21]. In the absence of broad methodological studies (e.g., via simulation approaches) in this field, we acknowledge that some of the choices we have done in presenting methods carry some amount of subjectivity and encourage the development of studies to quantitatively assess bias and trade-offs in this area to help investigators make more informed choices with regards to the methodological options. Such simulation studies could e.g., be used to select the most efficient approach to assess exposures in a given context.

To conclude, we will touch upon issues related to the terminology of risk assessment and HIA studies, the distinction between human-derived and animal-derived (toxicological) risk assessment studies, and research needs.

### Issues related to terminology

Studies aiming at characterizing the health and societal impact of policies or environmental factors are riddled with many different terminologies and acronyms. This diversity of acronyms encompasses some real differences in aims or methodology, but is also due to the convergence of various research and application streams. Indeed, as already mentioned, these studies originate from the epidemiological research stream related to the concept of population attributable fraction, which dates back to the 1950s [7], from the development of legal requirements for environmental impact assessment before the development of new policies, plans or programs (which progressively also encompassed health issues), and from the applied stream of chemical risk assessment based on “regulatory toxicology” approaches and the risk assessment logic outlined in the USA National Research Council originally published in 1983 and also known as the “red book [1, 2]. Three key expressions are used: 1) burden of disease; 2) risk assessment; 3) health impact assessment.

*Burden of disease studies* generally correspond to an assessment of the risk (e.g., in terms of attributable cases or DALYs) associated with a given disease in human populations, without referring to an exposure possibly causing the disease or to a policy aiming at limiting its impact. However, when used in relation with a factor or family of factors, then only the risk (or disease burden) associated with this factor is considered (e.g., in “environmental burden of disease”), so that there is no essential distinction anymore with what we have discussed here. In practice, health impact assessment is often used in relation with a policy or intervention likely to affect health, while environmental burden of disease is often used in the absence of explicit consideration of a policy or intervention (see below).

Regarding health impact assessment, a difficulty arises from the fact that much of the theory and examples of HIA studies has been published in the grey literature [10]. The term has most often been used to assess the potential impact of a public policy, plan, program or project not yet implemented [3]. HIA is defined by WHO as “a combination of procedures, methods and tools by which a policy, program or project may be judged as to its potential effects on the health of a population, and the distribution of those effects within the population”. In addition, the consideration of inequalities (i.e., considering the distribution of risk within a population rather than only its mean value) has been put forward as an essential part of HIAs, at least in principle [110]. Several distinctions exist within the field of HIAs, which refer to various notions and dimensions [10, 110, 111]. Some of these notions are at different levels and hence not mutually exclusive (for example some distinctions refer to the way health is conceptualized, other to the qualitative or quantitative nature of the study, others to the level of participation of the considered population), making it difficult to suggest a simple and unified terminology. A distinction between “broad focus” HIA studies, in which “a holistic model of health is used, democratic values and community participation are paramount and in which quantification of health impacts is rarely attempted” [10], and “tight focus” HIAs, based on epidemiology and toxicology and tending towards measurement and quantification, is sometimes done [10]. “Analytical HIA” is sometimes used synonymously to these “tight focus” HIAs. Harris-Roxas and Harris also quote distinctions such as between quantitative and qualitative HIAs, to those relying on “tight” or “broad” definitions of health, to HIAs of projects or policies [111]. What we have reviewed here is close (if not equivalent) to these “analytical”, “tight focus” or “quantitative” HIAs.

Such quantitative HIAs typically aim at answering a question about the future (“how is the planned policy expected to affect the health of the concerned population?”) while many risk assessment studies aim at answering a question about the present, and are sometimes presented as considering a single factor at a time (typically, “how many lung cancer cases would be avoided in this city if WHO air pollution guidelines were respected?”). We come back to these apparent differences in the Sect. " Assessing the impact of policies versus the impact of exposures".

As already stated, in practice, some HIA or “risk assessment” exercises fall short of providing a quantitative estimate of the risk, e.g., because of a lack of relevant dose response functions or support to collect missing information; they may for example stop at the step of hazard identification. It is, in a way, what happens with animal-based risk assessment studies.

### Animal-based risk assessment studies

It is important to recall that in addition to the approach based on human-derived dose–response functions that we described above exists a whole stream of research and applied studies relying on animal-based dose–response functions and so-called toxicological reference values. The core approach of the benchmark dose (BMDL) consists in identifying an exposure level corresponding to an effect considered small in an animal model (say, a 5% decrease in organ weight or 5% increase in the frequency of a disease) [112]. The lower confidence bound of the benchmark dose is then divided by an uncertainty factor (typically, 100), to take into account between-species and within-species differences in sensitivity, and this value is used as a “daily tolerable dose”, or compared to the exposure distribution in a specific human population. Therefore, this approach aims at identifying a range of doses under which, under certain assumptions, there would be no “appreciable adverse health effects” of exposure. The comparison of the estimated daily tolerable dose with the exposure distribution in the considered population allows to identify if a substantial share of the population is above this daily tolerable dose, and thus finds itself at exposure levels that cannot be deemed safe (a qualitative rather than quantitative statement about risk). For this reason, these risk assessment approaches based on animal-derived reference values or dose–response functions rather correspond to *safety assessment*: they can allow to state that, given its exposure distribution, a given human population is “safe”, or unlikely to suffer appreciable adverse health effects, with respect to a specific exposure (or set of exposures if the approach is used in the context of mixtures of exposures) and do not strictly correspond to risk assessment as we defined it, i.e., the estimation of a number of disease cases (risks) attributable to an exposure in a population. To limit ambiguity, it might be relevant either to use the expression “safety assessment” when referring to the so-called risk assessment studies relying on animal derived toxicological reference values, or to distinguish “animal-based risk assessment” (which implies between-species extrapolation) from “human-based risk assessment” (which does not).

### Assessing the impact of policies *versus* the impact of exposures

**Fig. 9:**
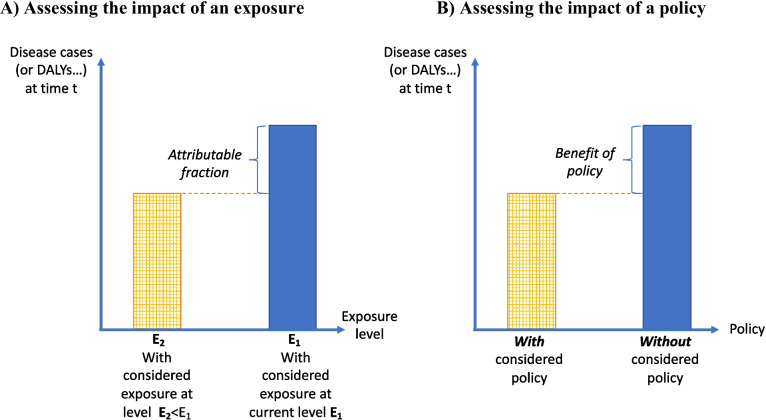
Illustration of the similitude of the principles of risk assessment of an exposure (**A**) and of a policy or program (**B**). When considering an exposure (**A**), the fraction of disease cases attributable to a specific exposure (compared to a lower and theoretically achievable level) is estimated for time t (typically assumed to correspond to the current time). When considering a policy (**B**), the expected health benefit of the project or policy (consisting in changing the level of one or more environmental factors) is estimated, considering the population at the current time or at a later time t, comparing it to the situation without change. Both approaches can be seen as aiming to estimate the impact of a theoretical policy or intervention lowering (or, more generally, changing) the level of one or several environmental factors, compared to a reference situation considered at the same time period

The factors influencing health considered in risk assessment studies range from single behaviors (e.g., smoking, physical activity), to physical and chemical factors such as particulate matter [35, 113] or other environmental exposures including lead or families of factors such as endocrine disruptors [12, 75]; these can be considered at various scales, from the neighborhood, region, country or at the planetary scale (e.g. as done by the Global Burden of Disease Studies, or by a study considering different ozone layer depletion scenarios [37]). In addition, as described above, the formalism of risk assessment studies used for a single exposure can be extended to the case of two or more exposures, at least under certain assumptions. Consequently, if one is interested in a project (e.g., the building of a road infrastructure, a factory) or a policy (regulating a behavior such as smoking, alcohol consumption, speed limit on highways, frequency of social contacts or exposure to a chemical or set of chemical factors, or consisting in taxes aiming at modifying exposures or behaviors), and if it is possible to provide a quantitative estimate of the expected changes in the factors affecting health impacted by this project or policy, then the methodology of risk assessment as described above can be used to provide an estimation of the impact of this project or policy. Symmetrically, evaluating the impact of a factor, such as an atmospheric pollutant, implies, as we have seen, to compare a given situation (usually, the current one or a future of this situation assuming a “business as usual” scenario) with a counterfactual situation (a hypothetical present in which a behavior or an exposure has been altered at the population level) that can be seen as resulting from a policy or an infrastructure. For example, assessing the risk associated with air pollution exposure implies to consider a counterfactual level (e.g., the WHO air pollution guidelines); the estimated risk is then identical to the health gain expected from a policy that would allow to lower air pollution from the current situation down to this guideline value (see Fig. 9 for an illustration). In other words, assessing ex-ante the impact of a hypothetical policy or infrastructure (what is sometimes termed health impact assessment) boils down to evaluating the health impact of its immediate consequences relevant for health (a set of factors); and assessing the impact of one or several behavioral, social or environmental factors (for which the expression risk assessment is usually reserved) is equivalent to considering the impact of a policy that would alter this or these factor(s). There may be some differences in implementation between the two specific questions (e.g., one may want to assume that it takes a few years for air pollution to reach the target value in the case of the evaluation of a policy, while an estimation of the current impact only requires to compare two “parallel worlds” with distinct air pollution levels), but these are not always considered and can be seen as minor technical differences. For these reasons, there are no essential differences in quantitatively assessing the effect of a single factor, of several factors, or of a policy or project. Recognizing this similarity in design of risk assessment and analytical/quantitative HIAs may allow to bring more clarity in the methodology and terminology; in particular, it may be relevant to adopt a unified terminology allowing to point to the differences that bear strong consequences, such as whether the study relies on human-based dose response functions (as illustrated here) or on dose–response functions derived from animal models.

### The perils of quantification: leaving emerging hazards by the roadside

Risk assessment studies imply many steps requiring a large amount of data; this is all the more true in the case of studies considering simultaneously multiple exposures, exposures with effects on multiple health endpoints (such as tobacco smoke, particulate matter, lead, physical activity…), or policies likely to influence several exposures or behaviors (such as a “zero pollution action plan”, as envisioned by the European Commission, or a set of actions to limit greenhouse gas emissions in multiple sectors). In some (probably not infrequent) cases, only a fraction of the data relevant for the risk assessment will be available or possibly available within a limited time frame. Researchers are then facing several non-exclusive options:

1. collect additional data (the scientifically rigorous approach); this may take a long time and be very expensive, since in many cases the missing data correspond to dose–response functions, which are typically generated by cohort studies. For example, in an ongoing exposome study conducted as part of ATHLETE project and considering 74 exposure-outcome pairs corresponding to effects with a level of evidence deemed likely or more than likely, a human dose–response function could be identified for only 70% of these possible effects (Rocabois et al., personal communication). Although working with high-quality data, even if this implies to delay the availability of the final results, is often the preferred option in science, such an option is problematic for health impact assessment studies, which often require to be conducted within a constrained time frame so that a decision about the planned policy or a possibly harmful exposure can be quickly taken, to bring potential health benefits to society or inform a legal process;
2. perform the study with the limited data available in the constrained time frame (imperfect but timely approach); in this case, it is possible that only a fraction of the impact of the exposure(s) or policy will be assessed (because several dose response functions corresponding to the effects of the exposure or policy are available) and that the quantified fraction will be estimated with large uncertainties;
3. perform a purely qualitative health impact assessment study (qualitative approach);
4. not to perform the study (“analysis paralysis”).

In many cases, option 2), consisting in moving along with the limited data available, will be preferred. The consequence may be that a fraction, which may be large, of the impact, will be ignored. Thus, because of their relative complexity, health impact assessment studies, which aim to make health impacts visible, may paradoxically leave a large fraction of this impact on the roadside. Impacts left on the road side will often correspond to “emerging” (newly identified factors, newly identified effects) risks. Under this imperfect but timely approach, it is essential not only to try to provide a quantification of the uncertainty around the quantified part (see above, Sect. " Sensitivity and uncertainty analyses"). It would also be relevant to attempt providing some estimate of the magnitude of what has obviously been left out (for example, the impact of a known exposure likely to affect an outcome for which no dose–response function is available), or at least to make the missing part (the “known unknown”) visible in some way.

### Identified gaps

This review provided a general methodological framework for risk assessment studies and demonstrated their relevance to also consider the expected impact of policies and infrastructures, and therefore their closeness to health impact assessment studies; it illustrated recent development related to the diversity of approaches to assess factors at the individual levels (such as fine-scale environmental models and personal dosimeters), and the potentially strong impact of choices regarding exposure assessment tools, including the consideration of population density when environmental models are used. It also allowed to identify some gaps, challenges or pending issues in the methodology of risk assessment studies. These issues include 1) proposing a formal approach to the quantitative handling of the level of evidence regarding each exposure-health outcome pairs (see Handling of the strength of evidence about the effect of environmental factors on health); 2) more generally, develop more formal and if possible quantitative assessment of the health impacts not handled by a specific quantitative risk assessment study (the “know unknowns”); 3) confronting the approaches of risk assessment based on human dose–response function reviewed here with that relying on toxicological data; and 4) other technical issues related to the simultaneous consideration of several exposures (or of policies acting on health via changes in several environmental factors), in particular when some of these exposures are causally related.

### Supplementary Information

**Additional file 1: Figure S1.**Illustration of the structure of a health impact assessment tool allowing quantification of the number of deaths preventable through compliance with recommendations regarding physical activity, air pollution, noise, heat and access to green space [36].

## Data Availability

Not applicable.
