# Supplementary material for: The methodology of quantitative risk assessment studies
Source: Environ Health. 2024 Jan 27;23:13. doi: 10.1186/s12940-023-01039-x (PMC10821313; doi:10.1186/s12940-023-01039-x)

Figure S1: Illustration of the structure of a health impact assessment tool allowing quantification of the number of deaths preventable through compliance with recommendations regarding physical activity, air pollution, noise, heat and access to green space [35].


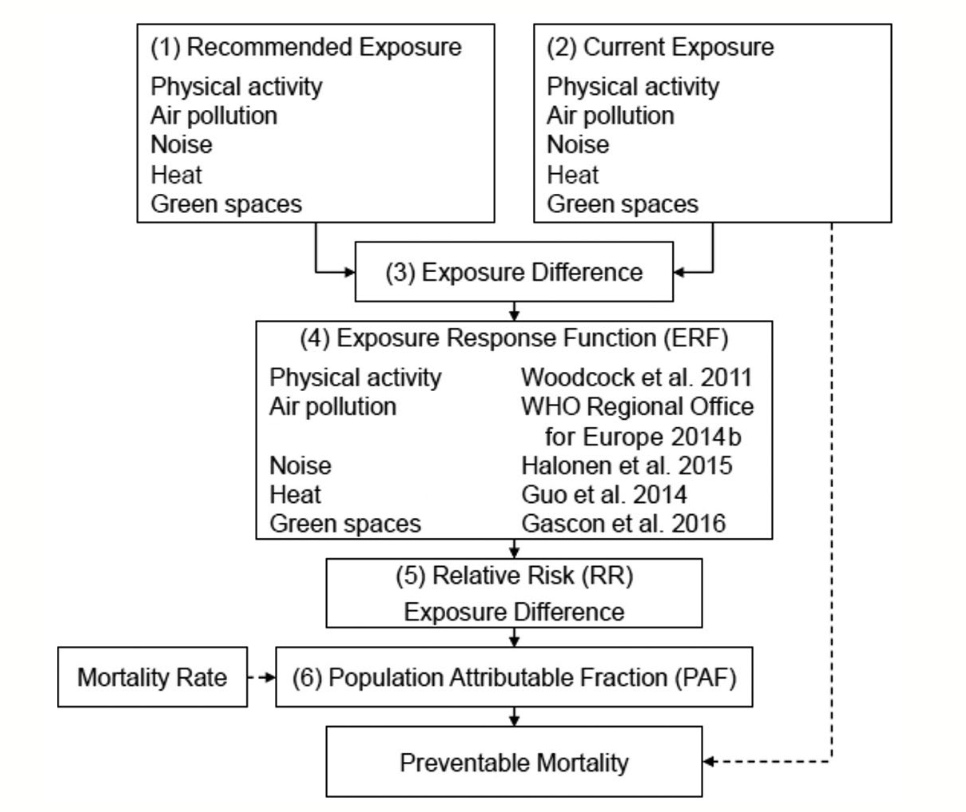

Supplement: Supplementary file 1 — Additional file 1: Figure S1.Illustration of the structure of a health impact assessment tool allowing quantification of the number of deaths preventable through compliance with recommendations regarding physical activity, air pollution, noise, heat and access to green space [36]. [file 12940_2023_1039_MOESM1_ESM.docx]
